# New Copper Complexes with N,O-Donor Ligands Based on Pyrazole Moieties Supported by 3-Substituted Acetylacetone Scaffolds

**DOI:** 10.3390/molecules29030621

**Published:** 2024-01-28

**Authors:** Jo’ Del Gobbo, Carlo Santini, Alessandro Dolmella, Zhenzhen Li, Miriam Caviglia, Maura Pellei

**Affiliations:** 1 School of Science and Technology, Chemistry Division, University of Camerino, Via Madonna delle Carceri (ChIP), Camerino, 62032 Macerata, Italy; jo.delgobbo@unicam.it (J.D.G.); carlo.santini@unicam.it (C.S.); zhenzhen.li@unicam.it (Z.L.); miriam.caviglia@unicam.it (M.C.); 2 Department of Pharmaceutical and Pharmacological Sciences, University of Padova, Via Marzolo 5, 35131 Padova, Italy

**Keywords:** copper, β-diketone, pyrazole, spectroscopy, X-ray

## Abstract

The new 3-monosubstituted acetylacetone ligands, 3-(phenyl(1*H*-pyrazol-1-yl)methyl)pentane-2,4-dione (HL^acPz^) and 3-((3,5-dimethyl-1*H*-pyrazol-1-yl)(phenyl)methyl)pentane-2,4-dione (HL^acPzMe^), were synthesized and used as supporting ligands for new copper(II) and copper(I) phosphane complexes of the general formulae [Cu(HL^acX^)_2_(L^acX^)_2_] and [Cu(PPh_3_)_2_(HL^acX^)]PF_6_ (X = Pz (pyrazole) or PzMe (3,5-dimethylpyrazole)), respectively. In the syntheses of the Cu(I) complexes, the triphenylphosphine coligand (PPh_3_) was used to stabilize copper in the +1 oxidation state, avoiding oxidation to Cu(II). All compounds were characterized by CHN analysis, ^1^H-NMR, ^13^C-NMR, FT-IR spectroscopy, and electrospray ionization mass spectrometry (ESI-MS). The ligands HL^acPz^ (**1**) and HL^acPzMe^ (**2**) and the copper complex [Cu(PPh_3_)_2_(HL^acPz^)]PF_6_ (**3**) were also characterized by X-ray crystallography. The reactivity of these new compounds was investigated and the new compounds 4-phenyl-4-(1*H*-pyrazol-1-yl)butan-2-one (**7**) and 4-(3,5-dimethyl-1*H*-pyrazol-1-yl)-4-phenylbutan-2-one (**8**) were obtained in basic conditions via the retro-Claisen reaction of related 3-monosubstituted acetylacetone, providing efficient access to synthetically useful ketone compounds. Compound **8** was also characterized by X-ray crystallography.

## 1. Introduction

β-diketone compounds represent a very important class of reagents in synthetic chemistry [1,2] with a well-established role in the synthesis of heterocyclic compounds [3,4,5]. The biological effects of these compounds are of great interest [6,7,8,9], and they have also been investigated as potential antiviral agents [10,11]. Although β-diketones represent one of the oldest classes of chelating ligands [12,13,14,15], their coordination chemistry continues to attract much interest, due to the ability of related metal complexes to support several unique and important catalytic reactions [16,17,18,19,20,21,22]. In this regard, it is often noted that even modestly sterically hindered β-diketones offer improvements over the parent acetylacetone [23]. The presence of steric bulk on β-diketones is of high interest, for their peculiar coordination behavior is useful to improve their catalytic activity and selectivity [24,25,26].

β-diketones are known to form complexes with almost every metal [16] and despite the enormous amount of work devoted to the synthesis and characterization of copper(II) β-diketonate complexes [27], there are relatively few reports devoted to the corresponding Cu(I) complexes showing undergoing disproportionation to copper metal and copper(II) compounds in the absence of stabilizing ligands [28,29,30]. In particular, very little attention has been paid to the study of triorganophosphane adducts of copper(I) β-diketonates of the general formula (β-diketonate)Cu(PR_3_)_n_, although a rich structural diversity can also be expected there [31,32,33,34,35,36,37,38,39,40,41,42,43]. On the other hand, very few studies on the biological activity of group 11 metal complexes of β-diketones have been reported in the literature to date [44] and copper(I)-based anticancer complexes supported by β-diketonate ligands remain an unexplored research field. Recently, as part of our continuous investigation on the chemical and biological properties of coinage metal complexes [45,46,47,48,49,50,51], we reported the first study on the syntheses, characterization and biological evaluation of new Cu(I) complexes containing triorganophosphanes and the anion of several sterically hindered the β-diketone ligands [52].

**Scheme 1:**
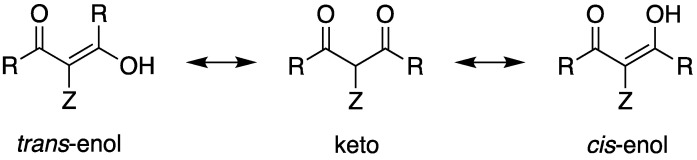
Keto-enol tautomeric equilibria.

On the other hand, α-monosubstituted β-diketones exhibit a range of reactivities depending on the nature of the substituents attached to the α-carbon atom [53]. The presence of the α-substituent significantly influences the behavior of these compounds, affecting their stability, reactivity, and reaction pathways [54]. There are three possible tautomeric isomers for symmetric and α-monosubstituted β-diketones (Scheme 1). Many factors influence the keto-enol tautomeric equilibrium [53,55], and among them it was found that electronegative β-substituents (R groups in Scheme 1) sharply increase the degree of enolization, whereas α-substituents (Z group in Scheme 1) cause a marked decrease in the percentage of enol [56]. However, the cause is mainly steric-repulsion between the α-moiety and the other β-substituents, which makes the chelate ring of the cis-enolic form unstable [55]. On the other hand, for 3-substituted-2,4-pentanediones, the keto form is predominant in the presence of bulky substituents in the 3-position [56]. 

Ditopic ligands based on 3-monosubstituted acetylacetone with two distinctly different coordination sites allow for assembling cations to coordination polymers, and thus add an additional degree of freedom to these complex solids [57,58,59,60,61]. Substituted acetylacetonates have often been used in this context and 3-ciano [62], 3-diazo [63], 3-pyridyl [64], 3-halogen [65], 3-caged phosphine [66], 3-(4-methylthiophenyl) [67] and 3-pyrazolyl [68] substituents, capable of supporting secondary interactions through exocyclic atoms with metal cations, have been described [57,64,69,70,71,72,73,74,75,76,77,78,79,80,81,82,83,84], and the subject has recently been reviewed [85].

**Scheme 2:**
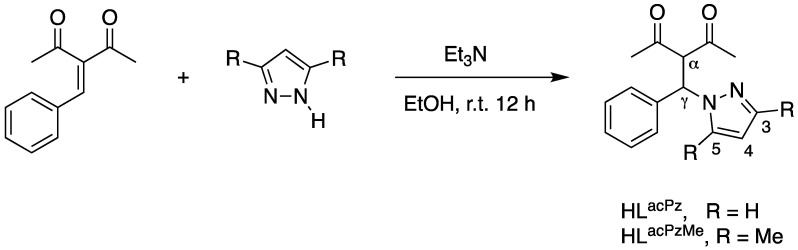
Synthesis of HL^acPz^ (**1**) and HL^acPzMe^ (**2**) ligands.

Therefore, as an extension of our studies on pyrazolyl derivatives and as part of our continuous investigation on the chemical and biological properties of copper-containing coordination compounds [46,47,48,49,52,86], we report here for the first-time a study on the syntheses, characterization and reactivity of new 3-monosubstituted acetylacetone ligands, HL^acPz^ and HL^acPzMe^ (Scheme 2), and related Cu(II) and Cu(I) complexes containing phosphane co-ligands, that could be evaluated for their biological effects and for their catalytic activity and selectivity. In particular, these species may be considered as versatile coordinating ligands having two different donor sites of different Pearson hardness [87] (a soft neutral nitrogen atom of the pyrazolyl moiety and a hard oxygen donor atoms of the acetylacetone moiety). In addition, in contrast to the aforementioned ditopic 3-monosubstituted acetylacetone ligands, HL^acPz^ and HL^acPzMe^ may act also as bidentate ligands at either the N or the O donor sites. In an endeavor to extend the range of metal chelates in which the β-diketones attain a conformation other than the usually encountered [85], herein we report the crystal structure of compound [Cu(HL^acPz^)(PPh_3_)_2_]PF_6_ that reveals the formation of a chelate complex, wherein the HL^acPz^ ligand coordinates in bidentate κ^2^*N,O* fashion to the copper(I) ion.

## 2. Results and Discussion

### 2.1. Synthesis and Characterization

The ligands HL^acPz^ (**1**) and HL^acPzMe^ (**2**) were prepared, using a modified literature method [88], from the reaction of 3-benzylidenepentane-2,4-dione and pyrazole or 3,5-dimethylpyrazole, respectively, in ethanol solution with triethylamine as basic catalyst, and isolated as white solids in very good yield and high purity (Scheme 2). The precursor 3-benzylidenepentane-2,4-dione can be synthetized by Knövenagel condensation of an equimolar amount of acetylacetone and benzaldehyde in the presence of piperidine as a catalyst [89,90].

Ligands **1** and **2** are soluble in methanol, diethyl ether, chloroform, acetonitrile, DMSO, THF, dichloromethane, ethyl acetate and acetone. They were fully characterized by multinuclear NMR spectroscopy, FT-IR, ESI-MS and elemental analysis. A batch of good quality crystals of **1** and **2**, suitable for X-ray analysis, was obtained by slow evaporation of a n-hexane and diethyl ether/n-hexane solution, respectively.

The IR spectra of **1** and **2** have been carried out on solid samples and show all the expected absorption bands. Weak bands in the region 2916–3108 cm^−1^ are assigned to C-H stretching vibrations. The ligands also show two sharp strong bands in the region 1699–1732 cm^−1^ attributable to the stretching vibrations of the two carbonyl groups. The bands at ca. 1500 cm^−1^ correspond to C=C/C=N rings stretching. The ^1^H-NMR spectra of HL^acPz^ and HL^acPzMe^ in CDCl_3_ suggest the presence of the keto form in solution. In the ^1^H-NMR spectrum of **1**, the 3-C*H* and 4-C*H* protons of pyrazole ring are present at 7.50 and 6.22 ppm, respectively. The 5-C*H* of pyrazole and the C*H* aromatic protons of the phenyl ring are present in the range 7.32–7.41 ppm. The α-C*H* and γ-C*H* protons are present at 5.32 (*J* = 11.28 Hz) and 6.01 ppm (*J* = 11.28 Hz). The singlets at 2.03 and 2.22 ppm are attributable to the methyl groups bonded to the β-dicarbonyl moieties. In the ^13^C-NMR spectrum in CDCl_3_, the *C*=O signals are detectable at about 200.0 ppm, while the α-*C*H and γ-*C*H carbons are visible at 72.6 and 64.2 ppm; the 4-*C*H of the pyrazole ring and the *C*H_3_ carbons are present at 106.1 and 29.9–30.9 ppm, respectively. In the ^1^H-NMR spectrum of **2** recorded in CDCl_3_ solution, the C*H* aromatic protons are present in the range 7.28–7.37 ppm and the α-C*H* and γ-C*H* proton are present at 5.82 ppm (*J* = 11.13 Hz) and 5.41 ppm (*J* = 11.11 Hz), respectively The singlets in the range 2.00–2.22 ppm are attributable to the methyl groups bonded to the β-dicarbonyl moieties and to the 3- and 5-C*H*_3_ of pyrazole, while the 4-C*H* proton is present as a singlet at 5.75 ppm. In the ^13^C-NMR spectrum, the *C*=O signals are detectable at 199.9 and 200.9 ppm, while the α- and γ-*C*H are at 72.4, and 60.6 ppm. The 4-*C*H carbon of the pyrazole ring is detectable at 105.6 ppm, while the 3- and 5-*C*H_3_ of the pyrazole and the *C*H_3_ of the β-dicarbonyl moieties are present at 10.9–13.6 and 30.3–31.6, respectively. The ESI-MS study has been performed by dissolving **1** and **2** in CH_3_CN and recording the spectra in the positive- and negative-ion modes. The molecular structure of **1** has been confirmed by the presence of the molecular peaks at *m*/*z* 257 and 279, respectively, attributable to the [HL^acPz^ + H]^+^ and [HL^acPz^ + Na]^+^ species. Analogously, in the positive-ion spectrum of **2** peaks at *m*/*z* 285 and 307, respectively, are attributable to the [HL^acPzMe^ + H]^+^ and [HL^acPzMe^ + Na]^+^ species. 

The Cu(I) complex [Cu(HL^acPz^)(PPh_3_)_2_]PF_6_ (**3**) was prepared from the reaction of PPh_3_, Cu(CH_3_CN)_4_PF_6_ and the ligand **1**, in CH_3_CN solution. Complex **3** is soluble in CHCl_3_, CH_3_CN, DMSO and acetone. Compound **3** was fully characterized by multinuclear NMR spectroscopy, FT-IR, ESI-MS and elemental analysis and a batch of good quality crystals, suitable for X-ray analysis, was obtained by slow evaporation of an acetone/ethyl acetate solution. The IR spectrum carried out on a solid sample of **3** shows all the expected bands for the β-diketone ligand and the triphenylphosphine co-ligand. The absorptions due to the C=O stretching are at 1687–1727 cm^−1^; they do not significantly vary with respect to the same absorptions of the carbonyl group detectable in the spectrum of the free ligand (1699–1732 cm^−1^). In a lower-frequency region, complex **3** shows a broad strong band at 831 cm^−1^ due to the stretching vibrations of the PF_6_^−^ anion. The δ(PF_6_) bending vibration is observed as a narrow strong band at 560 cm^−1^. In the ^1^H-NMR spectra of **3**, recorded in acetone-d_6_ solution at room temperature, the 3-C*H* and 4-C*H* protons of the pyrazole ring are present at 7.72 and 6.21 ppm, while the 5-C*H* proton of the pyrazole ring and the C*H* aromatic protons of the phenyl rings are present in the range 7.33–7.57 ppm. The α-C*H* and γ-C*H* bridging protons are present at 5.53 ppm (*J* = 11.40 Hz) and 6.10 ppm (*J* = 11.42 Hz). The singlets at 2.19 and 2.84 ppm are attributable to the methyl groups of the acetylacetone fragment; the signal at 2.84 is shifted with respect to the signal of the free ligand in the same solvent. In the ^13^C-NMR spectrum in acetone-d_6_, the *C*=O signals, are detectable at about 200 ppm. The ^31^P{H}-NMR spectrum of the Cu(I) complex **3**, recorded in CDCl_3_ solution at room temperature, gave a broad singlet at −0.86 ppm downfield shifted with respect to the value of the free phosphane (δ = −5.36 ppm). In the spectrum, the characteristic septet centered at −144.21 ppm is due to the PF_6_^−^ counterion. 

Compound [Cu(HL^acPzMe^)(PPh_3_)_2_]PF_6_^.^2CH_3_CN (**5**) was prepared similarly to compound **4** from the reaction of PPh_3_, Cu(CH_3_CN)_4_PF_6_ and the ligand **2**, in CH_3_CN solution and it was fully characterized. Complex **5** is soluble in CHCl_3_, CH_2_Cl_2_, CH_3_CN, ethyl acetate, THF, DMSO and acetone. The IR spectrum carried out on a solid sample of **5** shows all the expected bands for the β-diketone ligand and the triphenylphosphine co-ligand. The absorptions due to the C=O stretching are at 1700–1730 cm^−1^; they do not significantly vary with respect to the same absorptions of the carbonyl group detectable in the spectrum of the free ligand (1699–1729 cm^−1^). Very strong bands at 832 and 557 cm^−1^ are due to the stretching vibrations and the δ(PF_6_) bending vibrations, respectively, of the PF_6_^−^ anion. In the ^1^H-NMR spectra of **5**, recorded in CDCl_3_ solution at room temperature, the 3- and 5-C*H*_3_ protons of the pyrazole ring are present at 2.21 ppm, while the 4-C*H* proton of the pyrazole ring and the C*H* aromatic protons of the phenyl rings are present at 5.86 and at in the range 7.19–7.41 ppm, respectively. The α-C*H* and γ-C*H* bridging protons are present at 5.44 and 5.77 ppm. The singlets at 2.19 and 2.84 ppm are attributable to the methyl groups of the acetylacetone fragment; the signal at 2.84 is shifted with respect to the signal of the free ligand in the same solvent. In the ^13^C-NMR spectrum in CDCl_3_, the *C*=O signals, are detectable at about 200 ppm. The ^31^P{H}-NMR spectrum of **5**, recorded in CDCl_3_ solution at room temperature, gave a singlet at −0.12 ppm downfield shifted with respect to the value of the free phosphane. In the spectrum, the characteristic septet centered at −144.21 ppm is due to the PF_6_^−^ counterion. The ESI-MS study was performed by dissolving **3** and **5** in CH_3_CN and recording the spectra in the positive- and negative-ion modes. The molecular structure of **3** was confirmed by the presence of the peak at *m*/*z* 865, attributable to the molecular peak [Cu(L^acPz^)(PPh_3_)_2_ + Na]^+^; in addition, peaks at *m*/*z* 581 and 603 are attributable to the [Cu(HL^acPz^)(PPh_3_)]^+^ and [Cu(L^acPz^)(PPh_3_) + Na]^+^ species, being positive fragments of the dissociation of the PPh_3_ coligand from the complex. In both the compounds, the peaks at *m*/*z* 587 are due to the fragments of the dissociation of the ligand [Cu(PPh_3_)_2_]^+^. The ESI-MS spectra of **3** and **5** in CH_3_CN recorded in the negative-ion mode show peaks at *m*/*z* 145 attributable to the PF_6_^−^ aggregate, confirming the presence of the counterion (PF_6_^−^).

The copper(II) complexes [Cu(HL^acPz^)_2_(L^acPz^)_2_] (**4**) and [Cu(HL^acPzMe^)_2_(L^acPzMe^)_2_] (**6**) were prepared from the reaction of Cu(CH_3_CO_2_)_2_·H_2_O with HL^acPz^ (**1**) and HL^acPzMe^ (**2**), respectively, in methanol solution at room temperature, starting from a 1:4 stoichiometric ratio between metal and ligands. The syntheses were also performed starting from a 1:2 stoichiometric ratio obtaining the same products. The Cu(II) compounds **4** and **6** are soluble in Et_2_O, CH_2_Cl_2_, CHCl_3_, EtOAc, MeCN, DMSO, acetone and are air stable even as solutions; compound **6** is soluble in methanol too. The authenticity of compounds **4** and **6** was confirmed by elemental analysis, IR spectroscopy and electrospray mass spectra. The infrared spectra showed all the bands required by the presence of the chelating donors. In the spectra of compounds **4** and **6,** weak absorptions due to the CH stretching were observed at 2916–3107 cm^−1^. The carbonylic asymmetric stretching of **4** and **6** is detectable as strong absorptions at 1700–1734 cm^−1^, together with medium signals at 1667 and 1659 cm^−1^, respectively, in the typical range of β-diketonate systems [91]. The ESI-MS study was conducted by dissolving the Cu(II) complex compounds in acetonitrile, and recording the spectra in the positive- and negative-ion modes. In the positive-ion spectrum of **4,** it is possible to detect peaks at *m*/*z* 318 and 360 attributable to the [Cu(L^acPz^)]+ and [Cu(L^acPz^) + CH_3_CN]^+^ species, confirming the complex formation. 

**Scheme 3:**
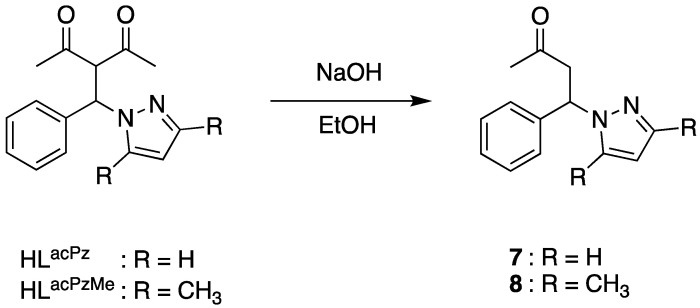
The Retro-Claisen reaction.

Both α-unsubstituted and α-monosubstituted β-diketones are well studied in retro-Claisen reactions [54,92,93,94,95,96,97]. The retro-Claisen reactions of β-diketones are extremely attractive, not only because these reactions are distributed widely in biological chemistry [7,98] but also because this C−C bond cleavage protocol provided efficient access to synthetically useful ester and ketone compounds, simultaneously [99,100,101,102]. In ethanol solution, HL^acPz^ and HL^acPzMe^ react with strong bases such as NaOH or t-BuOK by the retro-Claisen C−C bond cleavage reaction, giving rise to the formation of the species 4-phenyl-4-(1*H*-pyrazol-1-yl)butan-2-one, ^PhPz^MEK (**7**) and 4-(3,5-dimethyl-1*H*-pyrazol-1-yl)-4-phenylbutan-2-one, ^PhPzMe2^MEK (**8**) (Scheme 3). The reactions presumably proceed by the nucleophilic addition of bases to keto carbonyl group furnishing the key tetrahedral intermediate as a hemiketal anion, which might introduce C−C bond cleavage, leading to the retro-Claisen reaction, where an enolate anion intermediate is potentially involved in the reaction process [54].

The reaction was found to be highly dependent on the basicity of the base. Weak bases (such as Et_3_N) gave poor yield or even no reaction, while strong bases afforded a complete transformation. It is interesting to note that compound **7** was obtained in ethanol solution by the reaction of HL^acPz^ with strong bases only in small amounts, in the presence of unreacted ligand. Compounds **7** and **8** were fully characterized by multinuclear NMR spectroscopy, FT-IR, ESI-MS and elemental analysis and in addition a batch of good quality crystals of **8**, suitable for X-ray analysis, was obtained by slow evaporation of a diethyl ether/n-hexane solution.

The IR spectrum of **7** and **8** have been carried out on solid samples and show all the expected absorption bands. Weak bands in the region 2833–3106 cm^−1^ are assigned to C-H stretching vibrations, while the presence of only one strong band at about 1715 cm^−1^, due to the stretching vibration of the carbonyl group, confirms the deacetylation of **1** and **2** by the retro-Claisen reaction. In the ^1^H-NMR spectrum of **7** and **8** recorded in CDCl_3_ solution, the C*H* aromatic protons are present in the range 7.17–7.34, the singlets at 2.19 and 2.26 ppm are attributable to the methyl bonded to the carbonyl group, respectively in **7** and **8**. The C*H* bridging proton is present as a double doublet at 5.73–5.89 ppm. The diasterotopic protons of the methylene are detectable as double doublets at 3.05–3.11 ppm and 3.93–3.99 ppm. In the ^13^C-NMR spectra of **7** and **8**, recorded in CDCl_3_ the diagnostic *C*H_2_ signals, are detectable at 48.7 and 49.0 ppm, respectively. In the ESI-MS spectra of **7** and **8**, recorded in acetonitrile in the positive-ion mode, it is possible to detect the molecular peaks at *m*/*z* 215 and 243 attributable to the protonated species [^PhPz^MEK + H]^+^ and [^PhPzMe2^MEK + H]^+^, respectively.

### 2.2. X-ray Crystallography

**Figure 1:**
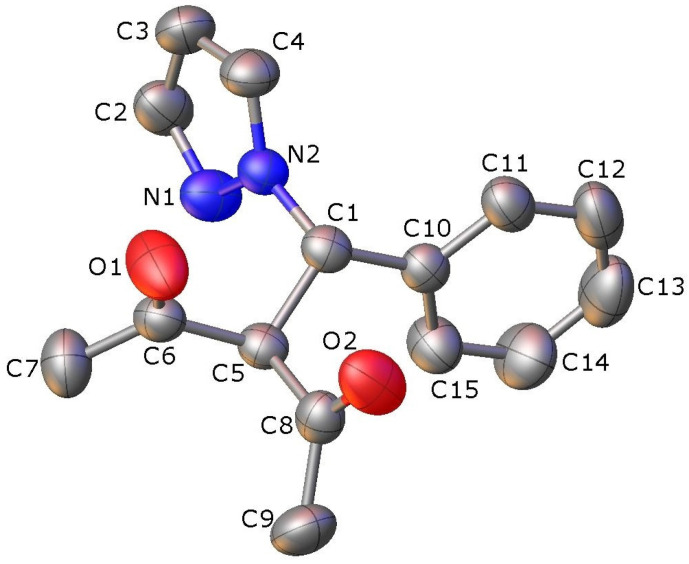
ORTEP representation of the HL^acPz^ ligand (**1**), with the atomic numbering scheme. Ellipsoids drawn at the 50% probability level; H atoms not shown.

**Figure 2:**
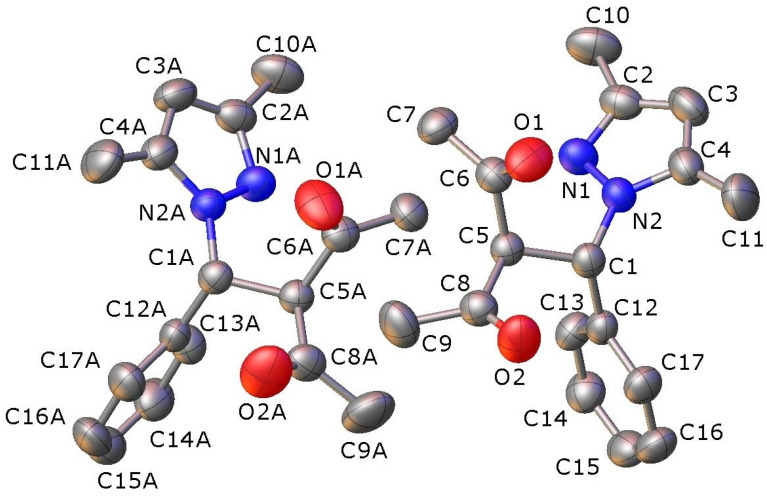
ORTEP representation of the two independent molecules in the asymmetric unit of the HL^acPzMe^ ligand (**2**), with the atomic numbering scheme. Ellipsoids drawn at the 50% probability level; H atoms not shown.

**Figure 3:**
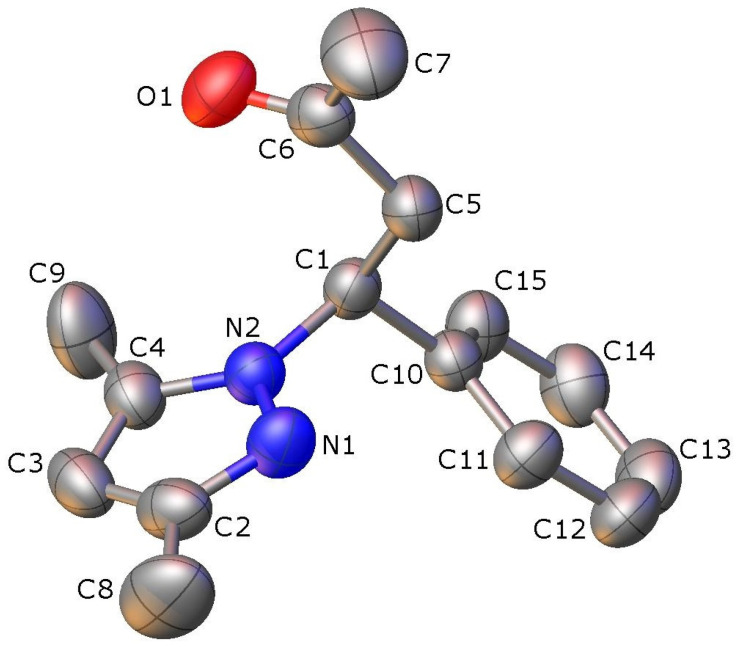
ORTEP representation of the ^PhPzMe2^MEK molecule (**8**), with the atomic numbering scheme. Ellipsoids drawn at the 50% probability level; H atoms not shown.

**Figure 4:**
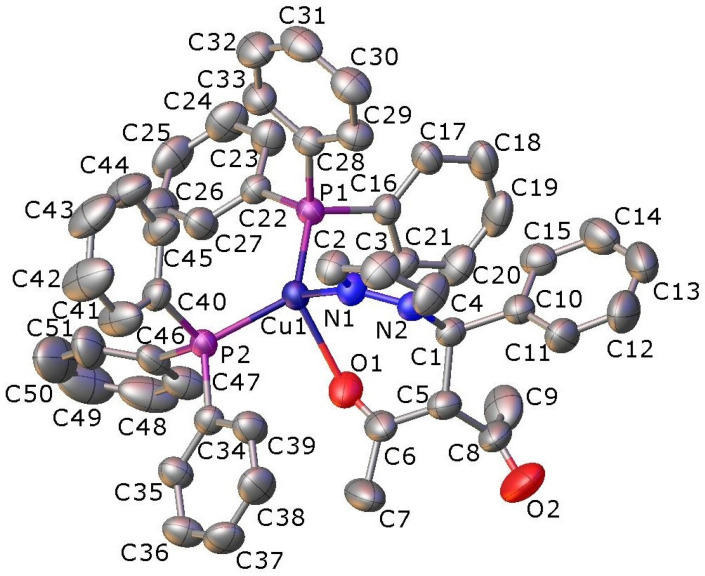
ORTEP representation of the [Cu(PPh_3_)_2_(HL^acPz^)]^+^ cation of the complex (**3**), with the atomic numbering scheme. Ellipsoids drawn at the 50% probability level; H atoms and the disordered PF_6_^−^ anion not shown.

A summary of the data collection parameters and crystal data for the HL^acPz^ (**1**) and HL^acPzMe^ (**2**) ligands, the ^PhPzMe2^MEK molecule (**8**) and the [Cu(PPh_3_)_2_(HL^acPz^)]PF_6_ complex (**3**) are reported in Table 1. Table 2, Table 3, Table 4 and Table 5 list a selection of bond lengths and angles of these compounds, while ORTEP [103] representations of the pertinent molecular structures are shown in Figure 1, Figure 2, Figure 3 and Figure 4. The selected specimens of **1**, **2** and **8** (Table 1) were all found to belong to the monoclinic crystal system; compound **8** was crystallized as a non-merohedral, two-component twin, with the two components having the same cell parameters. The twin data finalization showed that the whole reflection dataset was unequally divided into a major and a minor component (0.7761/0.2239 ratio). The *R*_int_ values for the two sets of reflections were significantly different, with the *R*_int_ value for the data of the minor component significantly worse. Accordingly, the molecular structure of **8** has been solved analyzing only data pertaining to the major component. Compounds **1**, **2** and **8** share a common 4-phenyl-4-(1H-pyrazol-1-yl)butan-2-one moiety and their structures can be discussed together. An examination of the CCDC repository [104,105] shows that the above fragment has not yet been documented, so, to the best of our knowledge, these molecules and complex **3** below are the first examples of structures containing this rather unusual synthon, while only few compounds show only some degree of similarity with those described here. Among these, ethyl N-(2-acetyl-3-oxo-1-phenylbutyl)carbamate [106] incorporates the 3-phenylmethyl-2,4-pentanedione moiety bound to a *sp*^2^ nitrogen that is also present in the **1** and **2** ligands, while few [107,108,109] bis-pyrazolyl derivatives show the pyrazole ring bound to a short alkyl-carbonyl chain. **1**, **2** and **8** are optically active molecules, because the C1 atom of all compounds shows four different substituent groups.

The X-ray experiments for **1** (Figure 1) and **8** (Figure 3) reveal that the asymmetric unit of these compounds is made of a single molecule, in which C1 has an *S* configuration. The asymmetric unit of compound **2** (Figure 2) shows instead two independent molecules, defining an enantiomeric pair, with C1 and C1A atoms showing, respectively, *S* and *R* configurations. The atoms common to **1**, **2** and **8** are reasonably superimposable (RMS value within 0.315–0.489 Å, making allowance for the inversion of the *R* enantiomer of **2**) by using the overlay routine in *Mercury* [110]. In **1** and **2**, the two -C(O)-CH_3_ groups branching from C5 have a nearly eclipsed reciprocal arrangement. In all compounds, the carbonyl oxygen atoms are always *syn*-placed with respect to the C1 methine hydrogen and the molecular pucker brings the mean planes encompassing the phenyl and the pyrazolyl moieties to form similar angles with each other; 73.8° in **1**, 71.8 and 67.5° in the two enantiomers of **2** and 61.6° in **8**. In the latter, the propan-2-one chain bound to C1 is also planar and makes angles of 87.7 and 54.1°, respectively, with the mean planes of the pyrazolyl and phenyl moieties. A comparison (Table 2, Table 3, Table 4 and Table 5) of the selected structural parameters in the common fragments of the three molecules indicate that the distribution of the bond lengths in the pyrazolyl rings and in the 2,4-pentanedione/propan-2-one chains is comparable, and it is also comparable with those reported for the few molecules [106,107,108,109] sharing some similarities with the compounds described here. In particular, the C=O bonds (mean length of 1.199 Å) have clear double bond character and the bonds made by the C1 atom (mean length of 1.526 Å) have single-bond character. Likewise, the distribution of the average bond lengths about the pyrazolyl rings (1.356, 1.332, 1.384, 1.368 and 1.465 Å for, respectively, N1-N2, N1-C2, C2-C3, C3-C4, N2-C4 distances) matches those found in reported [107,108,109] compounds (1.366, 1.334, 1.394, 1.367, 1.357 and 1.462 Å), thus suggesting that there is not conjugation outside the ring and that N1-C2 and C3-C4 distances have the greater double-bond character. As for the above compounds, the exploration of the CCDC database reveals only one bis-triphenylphosphine copper(I) complex having some similarity with the one presented here [111].

The complex **3** crystallizes in the orthorhombic crystal system; in its structure, the Cu(I) ion is surrounded by two unidentate PPh_3_ molecules and one bidentate HL^acPz^ neutral ligand, which attacks the copper ion by means of its N1, O1 atoms. The resulting complex [Cu(PPh_3_)_2_(HL^acPz^)]^+^ is cationic and crystallizes with the help of a (disordered) hexafluorophosphate anion. The central metal has a distorted tetrahedral environment (τ_4_ and τ_4′_ indexes values of 0.794 and 0.768, respectively [112,113]), dominated at one side by the large P1-Cu1-P2 angle of 128.25(4), to make allowance for the bulky triphenylphosphine ligands and at the other side by the narrow N1-Cu1-O1 chelation angle of 87.81(13). Notably, upon coordination, the HL^acPz^ ligand forms a seven-membered metallacycle, in which, in addition to C1, also C5 becomes optically active, with both centers having an *R* configuration. In its bound form, only the O6 atoms remains *syn*-oriented with respect to the methine C1 hydrogen, while O5 becomes *anti*-oriented. Accordingly, the two -C(O)-CH_3_ groups branching from C5 are also reciprocally *anti*-oriented with respect to the C5 atom. The atoms participating in the seven-membered ring, in addition to Cu1, O1 and N1, are N2, C1, C5 and C6. The ring has a distorted *boat* (*C*_s_) arrangement, due to the unequal Cu1-O1, Cu1-N1 distances as well as to the constraints imposed by the pyrazolyl ring. The Cu1, N1, O1 and C6 atoms are coplanar within 0.06 Å; another plane encompasses N1, N2, C5 and C6, coplanar within 0.02 Å, with C1 out of this last plane by 0.78 Å. The mean planes passing through the phenyl and the pyrazolyl rings in **3** make with each other a dihedral angle of 70.2° (compared with 73.8° in the free ligand), so the HL^acPz^ ligand satisfies the copper coordination needs by properly adjusting only its 2,4-pentanedionato moiety. The two triphenylphosphine ligands have the expected propeller shape, with the planes of the three phenyl rings making with each other angles of 64.2, 73.4, 88.1° for the rings branching from P1 and 78.1, 82.8, 87.3° for the rings branching from P2, respectively. Notably, the atoms of the C22/C27 ring, P1 and Cu1 are also coplanar within 0.03 Å. The C6-O1 and N1-N2 bond distances (Table 5) slightly elongate upon coordination (+0.008 and +0.006 Å, respectively), while the N1-C2 and C3-C4 distances shrink a bit, slightly increasing their double-bond character (−0.010 and −0.008 Å, respectively).

By examining the metrical data of the above noted (1H-pyrazole-3,5-dicarboxylato)-bis(triphenylphosphine)-copper(I) [111], we note that Cu-P distances in **3**, (2.2385(9)/2.2578(9) Å) compare quite well with those in the reported complex (2.226/2.265 Å) and are little shorter than the average of 2.272 Å for 950 bis-triphenylphosphine copper(I) complexes in the CCDC repository. On the other side, Cu-N (2.039(3) Å) and Cu-O (2.307(3) Å) distances are instead shorter and longer (−0.05 and +0.147 Å, respectively) than the corresponding ones [111] and also appreciably longer (+0.104 and +0.127 Å) of the known means for Cu(I) complexes showing Cu-N(pyrazolyl) and Cu-O=C bonds (1.935 Å/290 compounds and 2.180 Å/260 compounds, respectively). The longer Cu-N and Cu-O bonds (compared to averages for known compounds) may hint in part to a lesser donating ability of the N1 and especially O6 atoms, as well as to the steric hindrance brought by the bulky triphenylphosphine ligands. With respect to the nonbonding contacts, a list of the tightest interactions for the compounds described in this work is given in Table 6 (involved distances equal sum of the pertinent van der Waals radii minus 0.05 Å and almost all of them fall below 2.7 Å).

An inspection of the packing diagrams of the compounds **1** and **8** shows a similar contact network. In **1**, the two noticeable contacts involve O1 and N1 that binds, respectively the H15 and H11 atoms of two different nearby molecules. (at *x*, 1 − *y*, –1/2 *+ z* and *x*, 1 − *y*, 1/2 *+ z*). The two contacts help to consolidate a one-dimensional chain that propagates along the crystallographic *c* axis. In **8**, the three shortest contacts (range: 2.55–2.66 Å) all involve the O1 atom which binds, again, the H1/H15 and H14 of two different proximal molecules. The links with H1, H15 establish a dimeric couple, the third one (symmetry operation: 1 *+ x*, *y*, *z*) in turn propagates a one-dimensional chain of the dimer along the crystallographic *a* axis. In **2**, the two independent molecules in the asymmetric unit are bound through an O1A····H7B link (2.53 Å); O1A is also involved in a contact with the H14A of another molecule (at 1 *+ x*, *y*, *z*). Likewise, O1 establish two different contacts with the H7AC and H14 atoms 14 (at 1 *+ x*, *y*, *z* and −1 *+ x*, *y*, *z*) and all these links create again a one-dimensional chain propagating along the crystallographic *a* axis. This motif is also reinforced by an O2····H16 contact that joins together two proximal chains. The more extended nonbonding contact network of complex **3** depends on the disordered PF_6_^−^ anion. In the latter, almost all the atoms of the alternate position of the anion establish contacts with several hydrogen atoms sustaining a full 3D network in which the hexafluorophosphate anions and the complex cations pile up in alternate layers along the crystallographic *c* axis. Among these contacts, that between C15 and H37 of another molecule (at 1 − *x*, 1/2 − *y*, 1/2 *+ z*) of 2.79 Å is the longest one among those reported in Table 6 and also the only one that does not involve fluorine atoms; it creates a 1D chain propagating along the crystallographic *a* axis. Those involving F2 and F2A with H9B, F5A with H21 (all within the same unit) and F6A with H42 (at 1 − *x*, 1 − *y*, 1/2 *+ z*) range between 2.51 and 2.57 Å and sustain a zigzag 1D chain that propagates along the crystallographic *c* axis (Table 6). This chain is crisscrossed by another chain, running along the −1, 1, 0 plane and originated by the contacts of F1A with H14 and H18 (at1/2 *+ x*, 3/2 − *y*, *z*), F3 with H11, F4 with H5 and F4A with H38 (all of them at *x*, 1 *+ y*, *z*, range: 2.44–2.56 Å), which also intersects the motif originated by C15····H37 contact. The articulate 3D network is finally completed by the contact (2.63 Å) established between F3 and H32 (at 1/2 − *x*, 1/2 *+ y*, 1/2 *+ z*), which also intersects the aforementioned zigzag motif, keeping together two proximal zigzag chains. The nonbonding interactions above described are also illustrated with a series of crystal packing diagrams that have been attached to the Supporting Information (Figures S1–S8), highlighting the different structural motifs as well as the pertinent distances and angles.

## 3. Experimental Section

### 3.1. Materials and Instruments

All reagents were obtained from commercial suppliers and used as received. Melting Points (MP) were performed by an SMP3 Stuart Scientific Instrument (Bibby Sterilin Ltd., London, UK). Elemental Analyses (C, H, N, S) (EA) were performed with a Fisons Instruments EA-1108 CHNS-O Elemental Analyzer (Thermo Fisher Scientific Inc., Waltham, MA, USA). Fourier-Transform InfraRed (FT-IR) spectra were recorded from 4000 to 700 cm^−1^ on a PerkinElmer Frontier Instrument (PerkinElmer Inc., Waltham, MA, USA), equipped with the Attenuated Total Reflection (ATR) unit using universal diamond top-plate as sample holder. Abbreviation used in the analyses of the FT-IR spectra: br = broad, m = medium, mbr = medium broad, s = strong, sbr = strong broad, vs. = very strong, w = weak, and wbr = weak broad. Nuclear Magnetic Resonance (NMR) spectra for the nuclei ^1^H, ^13^C and ^31^P were recorded with a Bruker 500 Ascend Spectrometer (Bruker BioSpin Corporation, Billerica, MA, USA; 500.13 MHz for ^1^H, 125.78 MHz for ^13^C, 202.46 MHz for ^31^P and 470.59 MHz for ^19^F). Tetramethylsilane (SiMe_4_) was used as external standard for the ^1^H- and ^13^C-NMR spectra, while 85% H_3_PO_4_ was used for the ^31^P-NMR spectra. The chemical shifts (δ) are reported in ppm, and coupling constants (J) are reported in hertz (Hz). Abbreviation used in the analyses of the NMR spectra: br = broad, d = doublet, dbr = broad doublet, dd = doublet of doublets, m = multiplet, s = singlet, sbr = broad singlet, and t = triplet. Electrospray ionization mass spectra (ESI-MS) were recorded in the positive- (ESI-MS(+)) or negative-ion (ESI-MS(−)) modes on a Waters Micromass ZQ Spectrometer equipped with a single quadrupole (Waters Corporation, Milford, MA, USA), using methanol or acetonitrile mobile phase. The compounds were added to reagent grade methanol or acetonitrile to give approximately 0.1 mM solutions. These solutions were injected (1 µL) into the spectrometer fitted with an autosampler. The pump delivered the solutions to the mass spectrometer source at a flow rate of 200 μL/min and nitrogen was employed both as a drying and nebulizing gas. Capillary voltage was typically 2500 V. The temperature of the source was 100 °C, while the temperature of the desolvation was 400 °C. In the analyses of ESI-MS spectra, the confirmation of major peaks was supported by comparison of the observed and predicted isotope distribution patterns, the latter calculated using the IsoPro 3.1 computer software (T-Tech Inc., Norcross, GA, USA).

### 3.2. Synthesis

#### 3.2.1. Synthesis of HL^acPz^ (**1**)

Pyrazole (5.313 mmol, 0.362 g) was added to an ethanol solution (20 mL) of 3-benzylidene-2,4-pentadienone (5.313 mmol, 1.000 g) and triethylamine (6.206 mmol, 0.628 g) and the reaction was stirred overnight at reflux. The solution was left to cool to room temperature and dried at reduced pressure obtaining an orange oil product. The latter was solubilized in diethyl ether and precipitated with *n*-hexane to obtain the white solid product HL^acPz^ (**1**) in 70% yield. Crystals of ligand **1**, suitable for Single-Crystal X-ray Diffraction, were obtained by slow evaporation of a n-hexane solution of **1**. M.P.: 98–100 °C. FT-IR (cm^−1^): 3108w, 3056w (C-H); 1732s, 1699m (C=O); 1515w, 1505w, 1497w, 1454w, 1418m, 1394m, 1356s, 1323w, 1282sh, 1263s, 1231m, 1198m, 1168m, 1140s, 1096s, 1069w, 1046w, 1037w, 965m, 916w, 893m, 878w, 869w, 757vs, 729vs, 702s. ^1^H-NMR (CDCl_3_, 293 K): δ 2.03 (s, 3H, C*H*_3_), 2.22 (s, 3H, C*H*_3_), 5.32 (d, 1H, C*H*, *J* = 11.28 Hz), 6.01 (d, 1H, C*H*, *J* = 11.28 Hz), 6.22 (t, 1H, 4-C*H*_pz_, *J* = 2.06 Hz), 7.32–7.41 (m, 6H, C*H*_ar_ and 5-C*H*_pz_), 7.50 (dbr, 1H, 3-C*H*_pz_). ^1^H-NMR (DMSO, 293 K): δ 2.07 (s, 3H, C*H*_3_), 2.15 (s, 3H, C*H*_3_), 5.58 (d, 1H, C*H*, *J* = 11.55 Hz), 6.04 (d, 1H, C*H*, *J* = 11.28 Hz), 6.18 (t, 1H, 4-C*H*_pz_, *J* = 2.12 Hz), 7.26–7.50 (m, 6H, C*H*_ar_ and 5-C*H*_pz_), 7.85 (d, 1H, 3-C*H*_pz_, *J* = 2.12 Hz). ^1^H-NMR (Acetone-d_6_, 293 K): δ 2.07 (s, 3H, C*H*_3_), 2.18 (s, 3H, C*H*_3_), 5.51 (d, 1H, C*H*, ^3^*J* = 11.44 Hz), 6.08 (d, 1H, C*H*, ^3^*J* = 11.44 Hz), 6.19 (t, 1H, 4-C*H*_pz_, *J* = 2.06 Hz), 7.28–7.35 (m, 3H, C*H*_ar_), 7.44 (dbr, 1H, 5-C*H*_pz_), 7.51–7.53 (m, 2H, C*H*_ar_), 7.69 (d, 1H, 3-C*H*_pz_ *J* = 2.31 Hz). ^13^C{^1^H}-NMR (CDCl_3_, 293 K): δ 29.9, 30.9 (*C*H_3_), 64.2 (*C*H), 72.6 (*C*H), 106.1 (4-*C*H_pz_), 127.5, 128.7, 128.9, 129.6, 137.7, 139.4 (*C*H_ar_, 3- and 5-*C*H_pz_), 200.0 (*C*=O), 200.3 (*C*=O). ^13^C{^1^H}-NMR (Acetone-d_6_, 293 K): δ 29.5, 30.2 (*C*H_3_), 63.8 (*C*H), 71.4 (*C*H), 105.5 (4-*C*H_pz_), 127.8, 128.2, 128.5, 129.8, 138.7, 138.8 (*C*H_ar_, 3- and 5-*C*H_pz_), 199.5 (*C*=O), 199.8 (*C*=O). ESI-MS(+) (major positive ions, CH_3_CN), *m*/*z* (%): 211 (40) [3-benzylidene-2,4-pentanedione + Na]^+^, 257 (95) [HL^acPz^ + H]^+^, 279 (100) [HL^acPz^ + Na]^+^. ESI-MS(-) (major negative ions, CH_3_CN), *m*/*z* (%): 187 (100) [3-benzylidene-2,4-pentanedione − H]^−^, 213 (25) [HL^acPz^ − CH_3_CO]^−^. Elemental analysis (%) calculated for C_15_H_16_N_2_O_2_: C 70.29, H 6.29, N 10.93; found C 70.65, H 6.36, N 9.79.

#### 3.2.2. Synthesis of HL^acPzMe^ (**2**)

3,5-Methylpyrazole (5.313 mmol, 1.000 g) was added to an ethanol solution (20 mL) of 3-benzylidene-2,4-pentanedione (5.313 mol, 1.000 g) and triethylamine (6.206 mmol, 0.628 g), and the reaction was stirred overnight at room temperature. The solution was dried at reduced pressure obtaining a colorless oil. The latter was solubilized with diethyl ether and precipitated with *n*-hexane to obtain the white solid HL^acPzMe^ (**2**) in 73% yield. Crystals of ligand **2**, suitable for Single-Crystal X-ray Diffraction, were obtained by slow evaporation of a diethyl ether/n-hexane solution of **2**. M.P.: 89–92 °C. FT-IR (cm^−1^): 3034w, 2980w, 2916w (C-H); 1729s, 1699s (C=O); 1601w, 1586w, 1552m, 1496w, 1458m, 1422m, 1381m, 1350s, 1311w, 1294w, 1256sh, 1244s, 1234sh, 1192m, 1174m, 1134m, 1088w, 1071w, 1022m, 1002w, 972w, 951m, 930w, 898m, 864w, 853w, 813w, 776m, 763s, 717sh, 709vs. ^1^H-NMR (CDCl_3_, 293 K): δ 2.00, 2.18, 2.21, 2.22 (s, 12H, C*H*_3_), 5.41 (d, 1H, C*H*, *J* = 11.11 Hz), 5.75 (s, 1H, 4-C*H*_pz_), 5.82 (d, 1H, C*H*, *J* = 11.13 Hz), 7.28–7.37 (m, 5H, C*H*_ar_). ^13^C{^1^H}-NMR (CDCl_3_, 500 MHz): δ 10.9, 13.6, (3- and 5-*C*H_3_), 30.3, 31.6 (*C*H_3_), 60.6 (*C*H), 72.4 (*C*H), 105.6 (4-*C*H_pz_), 127.5, 128.2, 128.8, 129.0, 129.7, 138.0, 139.3, 147.3 (*C*H_ar_, 3- and 5-*C*_pz_), 199.9, 200.9 (*C*=O). ESI-MS(+) (major positive ions, CH_3_CN), *m*/*z* (%): 211 (50) [3-benzylidene-2,4-pentanedione + Na]^+^, 285 (100) [HL^acPzMe^ + H]^+^, 307 (45) [HL^acPzMe^ + Na]^+^. ESI-MS(−) (major negative ions, CH_3_CN), *m*/*z* (%): 187 (45) [3-benzylpentane-2,4-dione − H]^−^. Elemental analysis (%) calculated for C_17_H_20_N_2_O_2_: C 71.81, H 7.09, N 9.85; found C 73.45, H 7.29, N 9.70.

#### 3.2.3. Synthesis of [Cu(HL^acPz^)(PPh_3_)_2_]PF_6_ (**3**)

The ligand HL^acPz^ (1.000 mmol, 0.256 g) was added to an acetonitrile solution (40 mL) of PPh_3_ (2.000 mmol, 0.525 g) and Cu(CH_3_CN)_4_PF_6_ (1.000 mmol, 0.372 g) and stirred at room temperature for 12 h. The solvent was removed at reduced pressure, giving a yellow oil that was washed with diethyl ether, giving the white complex [Cu(HL^acPz^)(PPh_3_)_2_]PF_6_ in 89% yield. M.P.: 153–157 °C. Crystals of compound **3**, suitable for Single-Crystal X-ray Diffraction, were obtained by slow evaporation of an acetone/ethyl acetate solution of **3**. FT-IR (cm^−1^): 3063wbr (C-H); 1727w, 1687m (C=O); 1586w, 1480w, 1454w, 1435m, 1417w, 1363w, 1327w, 1279w, 1229w, 1194w, 1184w, 1172w, 1153m, 1120w, 1097m, 1070w, 1027w, 998w, 982w, 958w, 876w; 831vs (PF_6_); 787w, 750s, 729m, 695s. ^1^H-NMR (Acetone-d_6_, 293 K): δ 2.19 (s, 3H, C*H*3), 2.84 (s, 3H, C*H*3), 5.53 (d, 1H, C*H*, *J* = 11.40 Hz), 6.10 (d, 1H, C*H*, *J* = 11.42 Hz), 6.21 (t, 1H, 4-C*H*pz, *J* = 2.01 Hz), 7.33–7.57 (m, 36H, C*H*ar and 5-C*H*pz), 7.72 (d, 1H, 3-C*H*pz, *J* = 2.20 Hz). ^13^C{^1^H}-NMR (Acetone-d_6_, 293 K): δ 29.7, 30.4 (*C*H_3_), 64.2 (*C*H), 71.2 (*C*H), 106.2 (4-*C*H_pz_), 127.7, 128.4, 128.7, 128.9, 129.1, 129.7, 130.7, 131.2, 131.4, 133.5, 138.1; 199.6, 199.8 (*C*=O). ^31^P{^1^H}-NMR (CDCl_3_, 293 K): δ −0.86 (s), −144.21 (sept, *J*(^19^F-^31^P) = 712 Hz, PF_6_). ESI-MS(+) (major positive ions, CH_3_CN), *m*/*z* (%): 581 (30) [Cu(HL^acPz^)(PPh_3_)]^+^, 587 (100) [Cu(PPh_3_)_2_]^+^, 603 (30) [Cu(L^acPz^)(PPh_3_) + Na]^+^, 849 (10) [Cu(PPh_3_)_3_]^+^, 865 (5) [Cu(L^acPz^)(PPh_3_)_2_ + Na]^+^. ESI-MS(−) (major negative ions, CH_3_CN), *m*/*z* (%): 145 [PF_6_]^−^. Elemental analysis (%) calculated for C_51_H_46_CuF_6_N_2_O_2_P_3_: C 61.91, H 4.69, N 2.83; found: C 60.46, H 4.72, N 2.58. 

#### 3.2.4. Synthesis of [Cu(HL^acPz^)_2_(L^acPz^)_2_] (**4**) 

The ligand HL^acPz^ (4.000 mmol, 1.025 g) and copper(II) acetate monohydrate (Cu(CH_3_CO_2_)_2_·H_2_O (1.000 mmol, 0.199 g) were dissolved in CH_3_OH (40 mL), and the reaction was stirred for 12 h at room temperature. The mixture was filtered and the solution was dried at reduced pressure obtaining a blue oil. Diethyl ether was added, and the precipitate was filtered off. The solution was dried at reduced pressure to obtain the blue complex [Cu(HL^acPz^)_2_(L^acPz^)_2_] in 58% yield. M.P.: 87–88 °C. FT-IR (cm^−1^): 3107w, 3056w, 3031wbr, 3004wbr, 2916wbr (C-H); 1732s, 1700s 1657w (C=O); 1616w, 1517w, 1497w, 1453w, 141 8m, 1394 m, 1355s, 1322w, 1282sh, 1263s, 1230sh, 1917 m, 1168s, 1140s, 1095s, 1069w, 1045m, 1038m, 1030sh, 966m, 916w, 893m, 869w, 879w, 757s, 728s, 701s. ESI-MS(+) (major positive ions, CH3CN): *m*/*z* (%): 318 (30) [Cu(L^acPz^)]^+^, 360 (50) [Cu(L^acPz^) + CH_3_CN]^+^, 575 (10) [Cu(L^acPz^)_2_ + H]+. ESI-MS(−) (major negative ions, CH_3_CN): 255 (100) [L^acPz^]^−^. Elemental analysis (%) calculated for C_60_H_62_CuN_8_O_8_: C 66.31, H 5.75, N 10.31; found: C 67.84, H 6.14, N 9.18.

#### 3.2.5. Synthesis of [Cu(HL^acPzMe^)(PPh_3_)_2_]PF_6_^.^2CH_3_CN (**5**) 

The ligand HL^acPz^ HL^acPzMe^ (0.500 mmol, 0.142 g) was added to an acetonitrile solution (20 mL) of PPh_3_ (1.000 mmol, 0.262 g) and Cu(CH_3_CN)_4_PF_6_ (0.500 mmol, 0.186 g) and stirred at room temperature for 24 h. The solvent was removed at reduced pressure to obtain the white complex [Cu(HL^acPzMe^)(PPh_3_)_2_]PF_6_^.^2CH_3_CN in 73% yield. M.P.: 130–133 °C. FT-IR (cm^−1^): 3049w, 2988w, 2938w, 2902w (C-H); 2301w, 2271w (C≡N); 1730m, 1700m (C=O); 1667w, 1586w, 1554w, 1479m, 1458m, 1434s, 1394m, 1382m, 1331sh, 1311m, 1257m, 1246m, 1236sh, 1174mbr, 1160m, 1135m, 1121m, 1094s, 1067m, 1057m, 1027m, 1000m, 973w, 953w, 922vw, 898w, 879m, 859s; 832vs (PF_6_); 791m, 777m, 744vs, 693vs. 1H-NMR (CDCl_3_, 293 K): δ 2.06 (s, 6H, C*H*_3_), 2.21 (s, 12H, C*H*_3_), 5.44 (sbr, 1H, C*H*), 5.77 (sbr, 1H, C*H*), 5.86 (s, 1H, 4-C*H*_pz_), 7.19–7.41 (m, 35H, C*H*_ar_). ^13^C{^1^H}-NMR (CDCl_3_, 500 MHz): δ 1.98 (*C*H_3_CN), 11.0, 13.6 (3- and 5-*C*H_3_), 30.4, 31.5 (*C*H_3_); 60.6, 72.0 (*C*H); 105.8 (4-*C*H_pz_); 118.7 (CH_3_*C*N); 127.5, 128.3, 129.0, 129.7, 130.5, 131.1, 131.3, 132.0, 132.1, 133.4, 133.5, 137.8, 139.8, 147.6 (*C*H_ar_, 3- and 5-*C*_pz_); 200.1, 200.9 (*C*=O). ^31^P{^1^H}-NMR (CDCl_3_, 293 K): δ −0.12 (s), −144.23 (sept, *J*(^19^F-^31^P) = 712 Hz, PF_6_). ESI-MS(+) (major positive ions, CH_3_CN), *m*/*z* (%): 366 (70) [Cu(PPh_3_) + CH_3_CN]^+^, 589 (100) [Cu(PPh_3_)_2_]^+^. ESI-MS(−) (major negative ions, CH_3_CN), *m*/*z* (%): 145 (100) [PF_6_]^−^. Elemental analysis (%) calculated for C_57_H_56_CuF_6_N_4_O_2_P_3_: C 62.26, H 5.13, N 5.10; found: C 61.69, H 5.16, N 4.86.

#### 3.2.6. Synthesis of [Cu(HL^acPzMe^)_2_(L^acPzMe^)_2_] (**6**) 

The ligand HL^acPzMe^ (4.000 mmol, 1.137 g) and (Cu(CH_3_CO_2_)_2_·H_2_O (1.000 mmol, 0.199 g) were dissolved in CH_3_OH (40 mL), and the reaction was stirred for 24 h at room temperature. The solution was dried at reduced pressure obtaining a blue oil; diethyl ether was added and the precipitate was filtered off. The solution was dried at reduced pressure to obtain the blue complex [Cu(HL^acPzMe^)_2_(L^acPzMe^)_2_] in 50% yield. M.P.: 89–93 °C. FT-IR (cm^−1^): 3062w, 3031w, 3002wbr, 2919w (C-H); 1734s, 1703vs, 1659s (C=O); 1615m, 1556s, 1494m, 1455s, 1420s, 1382s, 1355vs, 1311m, 1288sh, 1245vs, 1213s, 1194sh, 1173s, 1086m, 1023m, 1005sh, 973m, 955m, 927m, 900m, 866m, 844w, 787m, 759s, 704vs. Elemental analysis (%) calculated for C_68_H_78_CuN_8_O_8_: C 68.12, H 6.56, N 9.35; found: C 69.89, H 6.80, N 9.16. 

#### 3.2.7. Synthesis of 4-Phenyl-4-(1H-pyrazol-1-yl)butan-2-one, ^PhPz^MEK (**7**)

An excess of sodium hydroxide (1.200 mmol, 0.048 g) was added to an ethanol solution (20 mL) of HL^acPz^ (1.000 mmol, 0.284 g), giving a pale-yellow solution. The reaction was stirred for 72 h at room temperature and the solution was dried at reduced pressure. The residue was washed with diethyl ether and hexane, the precipitate was filtered off and the solution was dried at reduced pressure, giving a yellow oil of ^PhPz^MEK. M.P.: oil. FT-IR (cm^−1^): 3106w, 3086w, 3061w, 3028w, 3003w, 2912w, 2833w (C-H); 1715s (C=O); 1659m, 1602m, 1510sh, 1494m, 1454m, 1435sh, 1417m, 1396s, 1358s, 1283m, 1255m, 1204m, 1160m, 1090m, 1044m, 1022m, 959m, 917m, 875m, 861m, 845m, 749vs, 697vs, 628sh, 618s. ^1^H-NMR (CDCl_3_, 293K): δ 2.19 (s, 3H, C*H*_3_), 3.11 (dd, 1H, C*H*_2_, *J* = 4.96 Hz, *J* = 17.28 Hz), 3.93 (dd, 1H, C*H*_2_, *J* = 9.05 Hz, *J* = 17.27 Hz), 5.89 (m, 1H, C*H*), 6.26 (s, 1H, 4-C*H*_pz_), 7.25–7.34 (m, 5H, C*H*_ar_), 7.43 (m, 1H, 5-C*H*_pz_), 7.56 (dbr, 1H, 3-C*H*_pz_). ^13^C{^1^H}-NMR (CDCl_3_, 500 MHz): δ 30.4 (*C*H_3_), 48.7 (*C*H_2_), 60.8 (*C*H), 105.7 (4-*C*H_pz_), 126.6, 128.0, 128.8, 129.7, 139.1, 140.5 (*C*H_ar_, 3- and 5-*C*_pz_), 205.2 (*C*=O). ESI-MS(+) (major positive ions, CH_3_CN), *m*/*z* (%): 215 (100) [^PhPz^MEK + H]^+^. Elemental analysis (%) calculated for C_13_H_14_N_2_O: C 74.35, H 7.49, N 11.56; found C, H, N.

#### 3.2.8. Synthesis of 4-(3,5-Dimethyl-1H-pyrazol-1-yl)-4-phenylbutan-2-one, ^PhPzMe2^MEK (**8**)

An excess of sodium hydroxide (1.200 mmol, 0.048 g) was added to an ethanol solution (20 mL) of HL^acPzMe^ (1.000 mmol, 0.284 g), giving a pale-yellow solution. The reaction was stirred for 12 h at room temperature and the solution was dried at reduced pressure. The residue was washed with chloroform, the precipitate was filtered off and the solution was dried at reduced pressure, giving an oil. The latter was solubilized in diethyl ether and n-hexane was added; white crystals of ^Ph PzMe2^MEK suitable for Single-Crystal X-ray Diffraction were obtained from a slow evaporation of the solution at a low temperature. M.P.: 98–100 °C. FT-IR (cm^−1^): 3123w, 3064w, 3008w, 2987w, 2947w, 2914w, 2898w, 2863w (C-H); 1714vs (C=O); 1601w, 1585w, 1552m, 1498m, 1459s, 1440m, 1425s, 1395s, 1370s, 1354s, 1323m, 1302w, 1274s, 1253m, 1200w, 1182m, 1162s, 1122w, 1060w, 1029s, 1018m, 1000w, 975m, 930w, 873m, 816w, 797vs, 775m, 762s, 710vs. ^1^H-NMR (CDCl_3_, 293 K): δ 2.19 (s, 6H, C*H*_3_), 2.26 (s, 3H, C*H*_3_), 3.05 (dd, 1H, C*H*_2_, *J* = 4.76 Hz, *J* = 17.31 Hz), 3.99 (m br, 1H, C*H*_2_), 5.73 (m, 1H, C*H*), 5.80 (s, 1H, 4-C*H*_pz_), 7.17–7.32 (m, 5H, C*H*_ar_). ^13^C{^1^H}-NMR (CDCl_3_, 500 MHz): δ 11.0, 13.6 (3- and 5-*C*H_3_), 30.6 (*C*H_3_), 49.0 (*C*H_2_), 56.9 (*C*H), 105.4 (4-*C*H_pz_), 126.3, 127.5, 128.7, 139.7, 141.0, 147.1 (*C*H_ar_, 3- and 5-*C*_pz_), 205.9 (*C*=O). ESI-MS(+) (major positive ions, CH_3_CN), *m*/*z* (%): 243 (100) [^PhPzMe2^MEK + H]^+^. Elemental analysis (%) calculated for C_15_H_18_N_2_O: C 74.35, H 7.49, N 11.56; found C 74.82, H 7.73, N 11.83.

### 3.3. Crystallographic Data Collection and Refinement

Single crystals suitable for the X-ray experiment of the compounds **1**, **2**, **8** were obtained by slow evaporation of *n*-hexane or diethyl ether/*n*-hexane solutions; likewise, crystals of the complex **3** were obtained by slow evaporation of an acetone/ethyl acetate solution. In all cases, several specimens were screened before selecting the most appropriate items, which were picked up with a nylon loop and mounted on the top of the goniometer head of a Rigaku-OD Gemini E diffractometer, equipped with a 2K × 2K EOS CCD area detector and sealed tube to enhance the Cu X–ray source. The raw diffraction data for all compounds were collected at room temperature [range: 298(1)–299.4(8) K] by means of the *ω*-scan technique, using graphite-monochromated Cu Kα radiation (λ = 1.54184 Å) in a 1024 × 1024-pixel mode and 2 × 2-pixel binning. Data collection, reduction, and finalization were performed using the *CrysAlisPro* software, Versions 1.171.41.123a (**1**) and 1.171.42.49 (**2**, **3**, **8**) [114]. Data collections were usually planned to allow for a certain degree of redundancy, except for compound **8**, where reflection intensities were low and the time for the experiment would have been exceedingly long. The raw data were corrected for Lorentz/polarization effects. An empirical absorption correction was also performed by means of a multiscan approach, with the scaling algorithm *SCALE*3 *ABSPACK*, using equivalent reflections. Accurate unit cell parameters were obtained by the least-squares refinement of 8464 (**1**), 19353 (**2**), 23975 (**3**), 2282 (**8**) strongest reflections chosen throughout the whole data collection. The crystal and equipment stability were checked by monitoring two reference frames every 50 frames for all compounds. A manual data reduction was performed at the end of the data collection in order to appropriately account for limited sample wobbling, but no significant change in peak intensities were observed during all experiments.

The structures were solved by direct phasing and refined by full-matrix least squares based on *F*_o_^2^ with the SHELXT [115] and SHELXL [116] programs through the OLEX2 program interface [117]. For all compounds, non-H atoms were allowed to vibrate anisotropically in the last cycles of refinement. In compounds **1**, **2** and **8**, the positions of the H atoms were obtained by difference Fourier maps; in complex **3**, H atoms were placed in calculated positions and refined as a riding model, with their displacement parameters calculated as 1.2 (or 1.5 for the methyl groups) times the *U*_eq_ of the pertinent carrier carbon atom. The asymmetric unit of compound **2** contains two independent molecules which define an enantiomeric pair, with C1 and C1A atoms showing, respectively, *S* and *R* configurations. The cationic complex **3** crystallizes as an hexafluorophosphate salt. The PF_6_^−^ anion is disordered over two positions, whose site occupation factors were constrained to sum to unity; finally refined sofs were 0.472/0.528 for F1/F6 and F1A/F6A, respectively. The involved atoms have also been modelled introducing RIGU restraints. The analysis of the diffraction data of **8** showed that this compound crystallizes in the form of a non-merohedral two-component twin (second component individuated by a rotation of -179.992° about the [1.00 0.00 0.00] direction in the reciprocal space). The two components account, respectively, for 77.61% and 22.39% of the diffraction peaks. Twin data finalization [114] of this compound showed that the *R*_int_ value of the data pertaining to the minor component was significantly worse than that of the major component; for this reason, the structure of **8** was solved using only the data of the major component. Full listings of atomic coordinates, bond lengths and angles, anisotropic thermal parameters are available as Supporting Information, in the form of .cif files; ORTEP [103] representations of all compounds (Figure 1, Figure 2, Figure 3 and Figure 4) have been prepared using the *Mercury* program [110]. CCDC 2326184 (**8**), 2326185 (**1**), 2326186 (**3**) and 2326187 (**2**) contain the supplementary crystallographic data for this paper, available free of charge from the Cambridge Crystallographic Data Centre via www.ccdc.cam.ac.uk/structures.

## 4. Conclusions

In this study, we report the synthesis and characterization of two new 3-monosubstituted acetylacetone ligands, 3-(phenyl(1*H*-pyrazol-1-yl)methyl)pentane-2,4-dione (HL^acPz^, **1**) and 3-((3,5-dimethyl-1*H*-pyrazol-1-yl)(phenyl)methyl)pentane-2,4-dione (HL^acPzMe^, **2**). They were employed for the preparation of the copper(II) and copper(I) phosphane complexes of the general formulae [Cu(PPh_3_)_2_(HL^acX^)]PF_6_ (**3** and **5**) and [Cu(HL^acX^)_2_(L^acX^)_2_] (**4** and **6**). HL^acPz^ and HL^acPzMe^ react with strong bases by the retro-Claisen C−C bond cleavage reaction, giving rise to the formation of the species 4-phenyl-4-(1*H*-pyrazol-1-yl)butan-2-one (**7**) and 4-(3,5-dimethyl-1*H*-pyrazol-1-yl)-4-phenylbutan-2-one (**8**), providing an efficient access to synthetically useful ketone compounds. All species were fully characterized both in the solid state and in solution. The X-ray experiments for **1** and **8** reveal that the related asymmetric unit is made of a single molecule, in which C1 has an *S* configuration. The asymmetric unit of compound **2** shows instead two independent molecules, defining an enantiomeric pair. The molecular structure reveals that complex **3** crystallizes in the orthorhombic crystal system, where copper(I) ion is surrounded in a distorted tetrahedral environment by two unidentate PPh_3_ molecules and one bidentate HL^acPz^ neutral ligand, which attacks the metal by means of its N1, O1 atoms. 

This study establishes a new and easily accessible class of copper compounds, provides new insights into the chemistry group 11 metal compounds, and opens new opportunities for further research in this field.

## Figures and Tables

**Table 1:** Summary of data collection parameters and crystal data for compounds **1**, **2**, **3** and **8**.

Compound	1	2	3	8
Radiation (all experiments)	Cu Kα (λ = 1.54184)			
Empirical formula	C_15_H_16_N_2_O_2_	C_17_H_20_N_2_O_2_	C_51_H_46_N_2_O_2_F_6_P_3_Cu	C_15_H_18_N_2_O
Formula weight	256.30	284.35	989.35	242.31
Temperature/K	298.1(4)	299.4(8)	298(1)	298.8(8)
Crystal system	monoclinic	monoclinic	orthorhombic	monoclinic
Space group	*C*2/*c*	*P*2_1_/*c*	*Pna*2_1_	*P*2_1_/*c*
*a*/Å	26.9711(5)	8.3223(2)	20.9587(2)	9.1203(7)
*b*/Å	9.7500(2)	12.4916(3)	9.96560(10)	12.8652(8)
*c*/Å	10.5745(2)	30.5285(5)	22.7132(2)	12.5423(9)
*α*/°	90.00	90.0	90.00	90.0
*β*/°	95.406(2)	91.296(2)	90.00	108.296(8)
*γ*/°	90.00	90.0	90.00	90.0
Volume/Å^3^	2768.39(9)	3172.90(12)	4744.02(8)	1397.25(18)
*Z*	8	8	4	4
ρ_calc_ Mg/m^3^	1.230	1.191	1.385	1.152
μ/mm^−1^	0.668	0.629	2.164	0.575
*F*(000)	1088	1216	2040	520
Crystal size/mm^3^	0.80 × 0.40 × 0.10	0.44 × 0.40 × 0.24	0.40 × 0.30 × 0.22	0.44 × 0.30 × 0.06
Reflections collected	20,260	53,329	41,569	5370
Independent reflections/*R*_int_	2658/0.0374	6320/0.0276	8023/0.0393	2484/0.0338
Restraints/parameters	0/237	0/539	1/643	0/236
Goodness of fit on *F*^2^	1.049	1.049	1.038	0.984
Final *R* (*R*_1_; *wR*_2_) indexes [*I* > 2σ (*I*)]	0.0473, 0.1113	0.0468, 0.1307	0.0336, 0.0838	0.0446, 0.1136
Largest diff. peak/hole/e Å^−3^	0.187/−0.175	0.222/−0.233	0.317/−0.182	0.149/−0.120

Goodness of fit = [Σ (w (F_o_^2^ – F_c_^2^)^2^]/(N_obsvns_ – N_params_)]^1/2^, based on all data; R_1_ = ||F_o_| – |F_c_||/|F_o_|; wR_2_ = [w (F_o_^2^ – F_c_^2^)^2^/w (F_o_^2^)^2^]^1/2^.

**Table 2:** Selected bond lengths (angstroms) and angles (°) for molecule **1**.

1			
Bond		Angle	
**N1-N2**	1.3412(18)	**N2-N1-C2**	103.65(14)
**N1-C2**	1.333(2)	**N1-N2-C4**	112.21(14)
**N2-C1**	1.4694(19)	**N1-N2-C1**	120.53(12)
**N2-C4**	1.338(2)	**N2-C1-C5**	109.98(12)
**C1-C5**	1.531(2)	**N2-C1-C10**	110.68(12)
**C1-C10**	1.520(2)	**C1-C5-C6**	110.1(9)
**C2-C3**	1.371(3)	**C1-C5-C8**	110.32(13)
**C3-C4**	1.359(3)	**C2-C3-C4**	104.79(16)
**C5-C6**	1.536(2)	**C5-C6-C7**	116.89(15)
**C5-C8**	1.532(2)	**C6-C5-C8**	107.35(12)
**C6-O1**	1.1953(19)	**C5-C6-O1**	120.18(15)
**C8-O2**	1.195(2)	**C5-C8-O2**	120.39(14)

**Table 3:** Selected bond lengths (angstroms) and angles (°) for molecule **2**.

2 *					
Bond			Angle		
**N1-N2**	1.3606(15)	1.3645(15)	**N2-N1-C2**	104.76(12)	104.73(11)
**N1-C2**	1.3293(17)	1.3298(17)	**N1-N2-C4**	112.45(11)	112.25(11)
**N2-C1**	1.4637(15)	1.4621(15)	**N1-N2-C1**	119.53(10)	119.45(10)
**N2-C4**	1.3513(17)	1.3496(17)	**N2-C1-C5**	109.65(10)	109.59(10)
**C1-C5**	1.5363(18)	1.5362(18)	**N2-C1-C12**	111.46(10)	112.36(10)
**C1-C12**	1.5213(18)	1.5211(18)	**C1-C5-C6**	111.23(10)	111.70(10)
**C2-C3**	1.388(2)	1.393(2)	**C1-C5-C8**	110.41(10)	110.27(11)
**C3-C4**	1.373(2)	1.366(2)	**C2-C3-C4**	106.70(13)	106.73(13)
**C5-C6**	1.5411(17)	1.5388(17)	**C5-C6-C7**	118.13(11)	117.63(12)
**C5-C8**	1.5380(17)	1.5342(17)	**C6-C5-C8**	107.02(10)	106.88(10)
**C6-O1**	1.2016(16)	1.2026(17)	**C5-C6-O1**	119.26(12)	119.57(13)
**C8-O2**	1.1964(18)	1.1948(19)	**C5-C8-O2**	120.77(13)	120.66(14)

* Compound **2** with two independent molecules in an asymmetric unit.

**Table 4:** **S**elected bond lengths (angstroms) and angles (°) for molecule **8**.

8			
Bond		Angle	
**N1-N2**	1.357(2)	**N2-N1-C2**	104.30(16)
**N1-C2**	1.334(3)	**N1-N2-C4**	112.47(15)
**N2-C1**	1.465(2)	**N1-N2-C1**	119.84(15)
**N2-C4**	1.352(2)	**N2-C1-C5**	109.70(15)
**C1-C5**	1.522(3)	**N2-C1-C10**	112.90(15)
**C1-C10**	1.518(2)	**C1-C5-C6**	113.97(17)
**C2-C3**	1.384(3)	**C2-C3-C4**	106.17(19)
**C3-C4**	1.372(3)	**C5-C6-C7**	116.7(2)
**C5-C6**	1.504(3)	**C5-C6-O1**	121.29(19)
**C6-O1**	1.211(2)		

**Table 5:** **S**elected bond lengths (angstroms) and angles (°) for complex **3**.

3			
Bond		Angle	
**Cu1-P1**	2.2385(9)	**N1-Cu1-P1**	110.40(9)
**Cu1-P2**	2.2578(9)	**N1-Cu1-P2**	109.52(10)
**Cu1-N1**	2.039(3)	**O1-Cu1-P1**	119.80(10)
**Cu1-O1**	2.307(3)	**O1-Cu1-P2**	93.11(9)
**N1-N2**	1.347(5)	**P1-Cu1-P2**	128.25(4))
**N1-C2**	1.323(5)	**N1-Cu1-O1**	87.81(13)
**N2-C4**	1.348(6)	**Cu1-P1-C16**	114.70(12)
**N2-C1**	1.470(5)	**Cu1-P2-C34**	117.08(12)
**C1-C5**	1.545(5)	**Cu1-N1-N2**	129.9(3)
**C5-C6**	1.525(6)	**Cu1-O1-C6**	136.9(3)
**C6-O1**	1.203(6)	**N1-N2-C1**	121.4(3)
**C5-C8**	1.551(7)	**N2-C1-C5**	110.9(3)
**C8-O2**	1.195(7)	**C1-C5-C6**	112.7(4)
**C2-C3**	1.369(6)	**C5-C6-O1**	122.1(4)
**C3-C4**	1.351(7)	**N1-N2-C4**	109.4(4)
**P1-C16**	1.833(4)	**N2-N1-C2**	105.8(3)
**P1-C22**	1.825(4)	**C16-P1-C28**	106.79(17)
**P1-C28**	1.828(4)	**C28-P1-C22**	102.83(18)
**P2-C34**	1.824(3)	**C34-P2-C46**	103.63(17)
**P2-C40**	1.829(4)	**C46-P2-C40**	106.5(2)
**P2-C46**	1.820(4)	**F-P3-F (av.)**	103.9
**P3-F (av.)**	1.540		

**Table 6:** Most efficient nonbonding interactions in molecules **1**, **2**, **8** (top), and complex **3** (bottom).

Molecule	Acceptor Atom (A) Atom	Donor (D) Atom	Parent (P) Atom *	Distance A····D (Å)	Angle A–D–P (°)	Symmetry Op.
**1**	O1	H15	C15	2.50	141.6	*x*, 1 *− y*, *−*1/2 *+ z*
	N1	H11	C11	2.70	171.6	*x*, 1 *− y*, 1/2 *+ z*
**2**	O1A	H7B	C7	2.53	147.0	*x*, *y*, *z*
	O1A	H14A	C14A	2.60	143.6	1 *+ x*, *y*, *z*
	O1	H7AC	C7A	2.52	156.0	1 *+ x*, *y*, *z*
	O1	H14	C14	2.56	152.3	*−*1 *+ x*, *y*, *z*
	O2	H16	C16	2.62	175.0	2 *− x*, 2 *− y,*1 *− z*
**8**	O1	H15	C15	2.55	153.4	1 *− x*, 1 *− y*, *−z*
	O1	H1	C1	2.65	157.3	1 *− x*, 1 *− y*, *−z*
	O1	H14	C14	2.66	136.6	1 *+ x*, *y*, *z*
**3**	F1A	H14	C14	2.56	135.3	1/2 *+ x*, 3/2 *− y*, *z*
	F1A	H18	C18	2.44	160.7	1/2 *+ x*, 3/2 *− y*, *z*
	F2A	H9B	C9	2.51	142.8	*x*, *y*, *z*
	F2	H9B	C9	2.56	130.7	*x*, *y*, *z*
	F2	H42	C42	2.44	145.6	1 *− x*, 1 *− y*, 1/2 *+ z*
	F3	H11	C11	2.51	158.2	*x*, 1 *+ y*, *z*
	F3	H32	C32	2.63	160.6	1/2 *− x*, 1/2 *+ y*, 1/2 *+ z*
	F4A	H38	C38	2.56	129.9	*x*, 1 *+ y*, *z*
	F4	H5	C5	2.46	154.5	*x*, 1 *+ y*, *z*
	F5A	H21	C21	2.57	148.4	*x*, *y*, *z*
	F6A	H42	C42	2.55	146.6	1 *− x*, 1 *− y*, 1/2 *+ z*
	C15	H37	C37	2.79	153.4	1 *− x*, 1/2 *− y*, 1/2 *+ z*

* Atom to which the donor atom is bound.

## Data Availability

The data presented in this study are available on request from the corresponding authors. CCDC 2326184 (**8**), 2326185 (**1**), 2326186 (**3**) and 2326187 (**2**) contain the supplementary crystallographic data for this paper, available free of charge from the Cambridge Crystallographic Data Centre via www.ccdc.cam.ac.uk/structures accessed on 27 December 2023.

## Supplementary Materials

The following supporting information can be downloaded at: https://www.mdpi.com/article/10.3390/molecules29030621/s1, Figures S1–S8: Crystal packing representation of compounds **1**, **2**, **3** and **8**; Figures S9–S33: FT-IR, ^1^H^−^, ^13^C^−^ and ^31^P-NMR spectra of compounds **1**–**8**.

## References

[1] Katritzky A.R., Wang Z., Wang M., Wilkerson C.R., Hall C.D., Akhmedov N.G. (2004). Preparation of β-keto esters and β-diketones by C-acylation/deacetylation of acetoacetic esters and acetonyl ketones with 1-acylbenzotriazoles. J. Org. Chem..

[2] Fargeas V., Baalouch M., Metay E., Baffreau J., Menard D., Gosselin P., Berge J.P., Barthomeuf C., Lebreton J. (2004). New access to 1,3-diketones from aldehydes. Tetrahedron.

[3] Sharma Y., Ansari A. (2022). Diketones as building block in organic synthesis with versatile applications and medicinal properties. Mater. Today Proc..

[4] Kaur N., Bhardwaj P., Gupta M. (2021). Recent Developments in the Synthesis of Five- and Six-membered N-heterocycles from Dicarbonyl Compounds. Curr. Org. Chem..

[5] Joule J.A., Mills K. (2010). Heterocyclic Chemistry.

[6] Hansen P.A.-O. (2021). Structural Studies of β-Diketones and Their Implications on Biological Effects. Pharmaceuticals.

[7] Grogan G. (2005). Emergent mechanistic diversity of enzyme-catalysed β-diketone cleavage. Biochem. J..

[8] Grogan G. (2002). β-diketone hydrolases. J. Mol. Catal. B Enzym..

[9] Pokorny D., Steiner W., Ribbons D.W. (1997). β-ketolases*-*Forgotten hydrolytic enzymes?. Trends Biotechnol..

[10] Hozakova L., Vokata B., Ruml T., Ulbrich P. (2022). Targeting the Virus Capsid as a Tool to Fight RNA Viruses. Viruses.

[11] Reina M., Talavera-Contreras L.G., Figueroa-DePaz Y., Ruiz-Azuara L., Hernández-Ayala L.F. (2022). Casiopeinas^®^ as SARS-CoV-2 main protease (M^pro^) inhibitors: A combined DFT, molecular docking and ONIOM approach. New J. Chem..

[12] Masui H. (2001). Metalloaromaticity. Coord. Chem. Rev..

[13] Siedle A.R., Wilkinson G., Gillard R.D., McCleverty J.A. (1987). Diketones and Related Ligands. Comprehensive Coordination Chemistry.

[14] Kawaguchi S. (1986). Variety in the coordination modes of β-dicarbonyl compounds in metal complexes. Coord. Chem. Rev..

[15] Mehrotra R.C., Bohra R., Gaur D.P. (1978). Metal [Beta]-Diketonates and Allied Derivatives.

[16] Crossman A.S., Marshak M.P., Constable E.C., Parkin G., Que L. (2021). 1.11-β-Diketones: Coordination and Application. Comprehensive Coordination Chemistry III.

[17] Primer D.N., Molander G.A. (2017). Enabling the Cross-Coupling of Tertiary Organoboron Nucleophiles through Radical-Mediated Alkyl Transfer. J. Am. Chem. Soc..

[18] Lo J.C., Gui J., Yabe Y., Pan C.M., Baran P.S. (2014). Functionalized olefin cross-coupling to construct carbon-carbon bonds. Nature.

[19] Shafir A., Buchwald S.L. (2006). Highly selective room-temperature copper-catalyzed C-N coupling reactions. J. Am. Chem. Soc..

[20] Baik T.G., Luis A.L., Wang L.C., Krische M.J. (2001). Diastereoselective cobalt-catalyzed Aldol and Michael cycloreductions. J. Am. Chem. Soc..

[21] Kang S.K., Lee S.H., Lee D. (2000). Copper-catalyzed N-arylation of amines with hypervalent iodonium salts. Synlett.

[22] Isayama S., Mukaiyama T. (1989). Hydration of Olefins with Molecular Oxygen and Triethylsilane Catalyzed by Bis(trifluoroacetylacetonato)cobalt(II). Chem. Lett..

[23] Stalpaert M., De Vos D. (2018). Stabilizing Effect of Bulky β-Diketones on Homogeneous Mo Catalysts for Deoxydehydration. ACS Sustain. Chem. Eng..

[24] Krajewski S.M., Crossman A.S., Akturk E.S., Suhrbier T., Scappaticci S.J., Staab M.W., Marshak M.P. (2019). Sterically encumbered β-diketonates and base metal catalysis. Dalton Trans..

[25] Crossman A.S., Larson A.T., Shi J.X., Krajewski S.M., Akturk E.S., Marshak M.P. (2019). Synthesis of Sterically Hindered β-Diketones via Condensation of Acid Chlorides with Enolates. J. Org. Chem..

[26] Akturk E.S., Scappaticci S.J., Seals R.N., Marshak M.P. (2017). Bulky β-Diketones Enabling New Lewis Acidic Ligand Platforms. Inorg. Chem..

[27] Gromilov S.A., Baidina I.A. (2004). Regularities of crystal structures of Cu(II) β-diketonates. J. Struct. Chem..

[28] Bulusheva L.G., Okotrub A.V., Liskovskaya T.I., Krupoder S.A., Guselnikov A.V., Manaev A.V., Traven V.F. (2001). Electronic Structure of 1,5-Cyclooctadiene-copper(I)-hexafluoroacetylacetonate. J. Phys. Chem. A.

[29] Chi K.M., Shin H.K., Hampden-Smith M.J., Duesler E.N., Kodas T.T. (1991). The chemistry of β-diketonate copper(I) compounds—III. The synthesis of (β-diketonate) Cu(1,5-COD) compounds, the solid state structure and disproportionation of hexafluoroacetylacetonato (1,5-cyclooctadiene)copper(I), (hfac)Cu(1,5-COD). Polyhedron.

[30] Nast R., Mohr R., Schultze C. (1963). Zur Kenntnis von Kupfer(I)-acetylacetonat. Chem. Berichte.

[31] Larson A.T., Crossman A.S., Krajewski S.M., Marshak M.P. (2020). Copper(II) as a Platform for Probing the Steric Demand of Bulky β-Diketonates. Inorg. Chem..

[32] Jakob A., Joubert C.C., Rüffer T., Swarts J.C., Lang H. (2014). Chemical and electrochemical oxidation studies on new copper(I) ferrocenyl-functionalised β-diketonates. Inorg. Chim. Acta.

[33] Lang H., Leschke M., Melter M., Walfort B., Kohler K., Schulz S.E., Gessner T. (2003). Mono- and bimetallic copper(I)- and silver(I)-phosphane complexes with β-diketonate units. Z. Anorg. Allg. Chem..

[34] Yang R.-N., Wang D.-M., Liu Y.-F., Jin D.-M. (2001). Synthesis of copper(I) β-diketone complexes. Polyhedron.

[35] Shin H.K., Chi K.M., Farkas J., Hampden-Smith M.J., Kodas T.T., Duesler E.N. (1992). Chemistry of copper(I).beta.-diketonate complexes. 2. Synthesis, characterization, and physical properties of (.beta.-diketonato)copper(I) trimethylphosphine and bis(trimethylphosphine) complexes. Inorg. Chem..

[36] Chi K.-M., Farkas J., Hampden-Smith M.J., Kodas T.T., Duesler E.N. (1992). The chemistry of copper(I)β-diketonate compounds. Part 4. Syntheses and characterization of CuXL(X = β-diketonate or Cl, L = PMe_3_, *n* = 2 or 4; L = PEt_3_, *n* = 2). Dalton Trans..

[37] Shin H.K., Hampden-Smith M.J., Duesler E.N., Kodas T.T. (1992). The chemistry of copper(I) β-diketonate compounds. Part V. Syntheses and characterization of (β-diketonate)CuL_n_ species where L = PBu_3_, PPh_3_, and PCy_3_; *n* = 1 and 2; crystal and molecular structures of (acac)Cu(PCy_3_), (tfac)Cu(PCy_3_), (hfac)Cu(PCy_3_), and (hfac)Cu(PCy_3_)_2_. Can. J. Chem..

[38] Baum T.H., Larson C.E. (1992). A novel copper complex and CVD precursor: (.eta.2-butyne)copper(I) hexafluoroacetylacetonate. Chem. Mater..

[39] Reynolds S.K., Smart C.J., Baran E.F., Baum T.H., Larson C.E., Brock P. (1991). Chemical vapor deposition of copper from 1,5-cyclooctadiene copper(I) exafluoroacetylacetonate. J. Appl. Phys. Lett..

[40] Beach D.B., LeGoues F.K., Hu C.K. (1990). Low-temperature chemical vapor deposition of high purity copper from an organometallic source. Chem. Mater..

[41] Chow Y.L., Buono-Core G.E. (1983). Triplet-state benzophenone-sensitized photoreduction of bis(acetylacetonato)copper(II): The generation and stability of copper(I) complexes. Can. J. Chem..

[42] Restivo R.J., Costin A., Ferguson G., Carty A.J. (1975). Perchlorato, Nitrato, and Acetylacetonato Complexes of Copper(I). The Crystal and Molecular Structure of Perchloratobis(tricyclohexyiphosphine)Copper(I). Can. J. Chem..

[43] Anderson W.A., Carty A.J., Palenik G.J., Schreiber G. (1971). Nitrato and Acetylacetonato Complexes of Copper(I). Can. J. Chem..

[44] Khamidullina L.A., Puzyrev I.S., Burygin G.L., Dorovatovskii P.V., Zubavichus Y.V., Mitrofanova A.V., Khrustalev V.N., Timofeeva T.V., Slepukhin P.A., Tobysheva P.D. (2021). Unsymmetrical Trifluoromethyl Methoxyphenyl β-Diketones: Effect of the Position of Methoxy Group and Coordination at Cu(II) on Biological Activity. Molecules.

[45] Pellei M., Santini C., Bagnarelli L., Caviglia M., Sgarbossa P., De Franco M., Zancato M., Marzano C., Gandin V. (2023). Novel Silver Complexes Based on Phosphanes and Ester Derivatives of Bis(pyrazol-1-yl)acetate Ligands Targeting TrxR: New Promising Chemotherapeutic Tools Relevant to SCLC Management. Int. J. Mol. Sci..

[46] Del Bello F., Pellei M., Bagnarelli L., Santini C., Giorgioni G., Piergentili A., Quaglia W., Battocchio C., Iucci G., Schiesaro I. (2022). Cu(I) and Cu(II) Complexes Based on Lonidamine-Conjugated Ligands Designed to Promote Synergistic Antitumor Effects. Inorg. Chem..

[47] Pellei M., Bagnarelli L., Luciani L., Del Bello F., Giorgioni G., Piergentili A., Quaglia W., De Franco M., Gandin V., Marzano C. (2020). Synthesis and Cytotoxic Activity Evaluation of New Cu(I) Complexes of Bis(pyrazol-1-yl) Acetate Ligands Functionalized with an NMDA Receptor Antagonist. Int. J. Mol. Sci..

[48] Morelli M.B., Amantini C., Santoni G., Pellei M., Santini C., Cimarelli C., Marcantoni E., Petrini M., Del Bello F., Giorgioni G. (2018). Novel antitumor copper(ii) complexes designed to act through synergistic mechanisms of action, due to the presence of an NMDA receptor ligand and copper in the same chemical entity. New J. Chem..

[49] Gandin V., Ceresa C., Esposito G., Indraccolo S., Porchia M., Tisato F., Santini C., Pellei M., Marzano C. (2017). Therapeutic potential of the phosphino Cu(I) complex (HydroCuP) in the treatment of solid tumors. Sci. Rep..

[50] Tisato F., Marzano C., Peruzzo V., Tegoni M., Giorgetti M., Damjanovic M., Trapananti A., Bagno A., Santini C., Pellei M. (2016). Insights into the cytotoxic activity of the phosphane copper(I) complex Cu(thp)_4_PF_6_. J. Inorg. Biochem..

[51] Papini G., Bandoli G., Dolmella A., Gioia Lobbia G., Pellei M., Santini C. (2008). New homoleptic carbene transfer ligands and related coinage metal complexes. Inorg. Chem. Commun..

[52] Pellei M., Del Gobbo J., Caviglia M., Karade D.V., Gandin V., Marzano C., Noonikara Poyil A., Dias H.V.R., Santini C. (2023). Synthesis and cytotoxicity studies of Cu(I) and Ag(I) complexes based on sterically hindered β-diketonates with different degrees of fluorination. Dalton Trans..

[53] Emsley J., Emsley J., Ernst R.D., Hathaway B.J., Warren K.D. (1984). The composition, structure and hydrogen bonding of the β-diketones. Complex Chemistry.

[54] Liu J.-H., Lv X.-J., Liu Y.-K. (2023). Asymmetric Retro-Claisen Reaction Catalyzed by Chiral Aza-Bisoxazoline–Zn(II) Complex: Enantioselective Synthesis of α-Arylated Ketones. Org. Lett..

[55] Kol’tsov A.I., Kheifets G.M. (1971). Investigation of Keto–Enol Tautomerism by Nuclear Magnetic Resonance Spectroscopy. Russ. Chem. Rev..

[56] Tanaka M., Shono T., Shinra K. (1969). Tautomerism in 3-Substituted-2,4-pentanediones and Their Copper Chelates. Bull. Chem. Soc. Jpn..

[57] Tong J., Jia L.-M., Shang P., Yu S.-Y. (2019). Controlled Synthesis of Supramolecular Architectures of Homo- and Heterometallic Complexes by Programmable Self-Assembly. Cryst. Growth Des..

[58] Lee E., Seo S.J., Lee S.S., Lindoy L.F. (2017). Assembling latter d-block heterometal coordination polymers: Synthetic strategies and structural outcomes. Coord. Chem. Rev..

[59] Cook T.R., Stang P.J. (2015). Recent Developments in the Preparation and Chemistry of Metallacycles and Metallacages via Coordination. Chem. Rev..

[60] Li H., Yao Z.J., Liu D., Jin G.X. (2015). Multi-component coordination-driven self-assembly toward heterometallic macrocycles and cages. Coord. Chem. Rev..

[61] Ward M.D., Raithby P.R. (2013). Functional behaviour from controlled self-assembly: Challenges and prospects. Chem. Soc. Rev..

[62] Silvernail C.M., Yap G., Sommer R.D., Rheingold A.L., Day V.W., Belot J.A. (2001). An effective synthesis of alkyl β-cyano-α,γ-diketones using chlorosulfonylisocyanate and a representative Cu(II) complex. Polyhedron.

[63] Nieuwenhuyzen M., Schobert R., Hampel F., Hoops S. (2000). Reactions of dicarbonyltitanocenes with 2-diazo-1,3-diketones: *O*-,*N*-versus O-,O-chelation and self-assembly of a novel heteroleptic Ti_5_O_6_-cage compound. Inorg. Chim. Acta.

[64] Turner S.S., Collison D., Mabbs F.E., Halliwell M. (1997). Preparation, magnetic properties and crystal structure of bis 3-(4-pyridyl)pentane-2,4-dionato copper(II). J. Chem. Soc. Dalton Trans..

[65] Isakova V.G., Baidina I.A., Morozova N.B., Igumenov I.K. (2000). gamma-Halogenated iridium(III) acetylacetonates. Polyhedron.

[66] Gildenast H., Hempelmann G., Gruszien L., Englert U. (2023). A Rigid Linker for Site-Selective Coordination of Transition Metal Cations: Combining an Acetylacetone with a Caged Phosphine. Inorg. Chem..

[67] Gildenast H., Nölke S., Englert U. (2020). 3-(4-Methylthiophenyl)acetylacetone–ups and downs of flexibility in the synthesis of mixed metal–organic frameworks. Ditopic bridging of hard and soft cations and site-specific desolvation. CrystEngComm.

[68] Guo Q.Q., Englert U. (2016). An Acetylacetonate or a Pyrazole? Both! 3-(3,5-Dimethyl-pyrazol-4-yl)pentane-2,4-dione as a Ditopic Ligand. Cryst. Growth Des..

[69] van Terwingen S., Nachtigall N., Ebel B., Englert U. (2021). N-Donor-Functionalized Acetylacetones for Heterobimetallic Coordination Polymers, the Next Episode: Trimethylpyrazoles. Cryst. Growth Des..

[70] Guo Q.Q., Merkens C., Si R.Z., Englert U. (2015). Crosslinking of the Pd(acacCN)_2_ building unit with Ag(I) salts: Dynamic 1D polymers and an extended 3D network. Crystengcomm.

[71] Merkens C., Truong K.N., Englert U. (2014). 3-(4-Pyridyl)-acetylacetone*-*a fully featured substituted pyridine and a flexible linker for complex materials. Acta Crystallogr. Sect. B Struct. Sci. Cryst. Eng. Mater..

[72] Merkens C., Pecher O., Steuber F., Eisenhut S., Gorne A., Haarmann F., Englert U. (2013). Crystal-to-Crystal Transformations in a Seven-Coordinated Scandium Complex. Z. Anorg. Allg. Chem..

[73] Merkens C., Pan F.F., Englert U. (2013). 3-(4-Pyridyl)-2,4-pentanedione*-*a bridge between coordinative, halogen, and hydrogen bonds. Crystengcomm.

[74] Merkens C., Englert U. (2012). Ordered bimetallic coordination networks featuring rare earth and silver cations. Dalton Trans..

[75] Merkens C., Becker N., Lamberts K., Englert U. (2012). Bimetallic coordination networks based on Al(acacCN)_3_: A building block between inertness and lability. Dalton Trans..

[76] Kondracka M., Englert U. (2008). Bimetallic Coordination Polymers via Combination of Substitution-inert Building Blocks and Labile Connectors. Inorg. Chem..

[77] Zhang Y.X., Chen B.L., Fronczek F.R., Maverick A.W. (2008). A nanoporous Ag-Fe mixed-metal-organic framework exhibiting single-crystal-to-single-crystal transformations upon guest exchange. Inorg. Chem..

[78] Burrows A.D., Cassar K., Mahon M.F., Warren J.E. (2007). The stepwise formation of mixed-metal coordination networks using complexes of 3-cyanoacetylacetonate. Dalton Trans..

[79] Vreshch V.D., Lysenko A.B., Chernega A.N., Sieler J., Domasevitch K.V. (2005). Heterobimetallic Cd(Zn)/Be coordination polymers involving pyridyl functionalized beryllium diketonates. Polyhedron.

[80] Chen B.L., Fronczek F.R., Maverick A.W. (2004). Porous Cu-Cd mixed-metal-organic frameworks constructed from Cu(PyaC)_2_ {Bis 3-(4-pyridyl)pentane-2,4-dionato copper(II)}. Inorg. Chem..

[81] Vreshch V.D., Lysenko A.B., Chernega A.N., Howard J.A.K., Krautscheid H., Sieler J., Domasevitch K.V. (2004). Extended coordination frameworks incorporating heterobimetallic squares. Dalton Trans..

[82] Vreshch V.D., Chernega A.N., Howard J.A.K., Sieler J., Domasevitch K.V. (2003). Two-step construction of molecular and polymeric mixed-metal Cu(Co)/Be complexes employing functionality of a pyridyl substituted acetylacetonate. Dalton Trans..

[83] Boldog I., Rusanov E.B., Chernega A.N., Sieler J., Domasevitch K.V. (2001). Acentric extended solids by self assembly of 4,4′-bipyrazolyls. Angew. Chem. Int. Ed. Engl..

[84] Mackay L.G., Anderson H.L., Sanders J.K.M. (1992). A platinum-linked cyclic porphyrin trimer. J. Chem. Soc. Chem. Commun..

[85] Kremer M., Englert U. (2018). N Donor substituted acetylacetones–versatile ditopic ligands. Z. Für Krist. Cryst. Mater..

[86] Pellei M., Papini G., Trasatti A., Giorgetti M., Tonelli D., Minicucci M., Marzano C., Gandin V., Aquilanti G., Dolmella A. (2011). Nitroimidazole and glucosamine conjugated heteroscorpionate ligands and related copper(II) complexes. Syntheses, biological activity and XAS studies. Dalton Trans..

[87] Pearson R.G. (1997). Chemical Hardness.

[88] Maier T., Mildenberger H. (1980). β-Azolyl-α,α-dicarbonyl compounds. Angew. Chem. Int. Ed. Engl..

[89] Raman N., Pravin N. (2014). Lasing the DNA fragments through β-diketimine framed Knoevenagel condensed Cu(II) and Zn(II) complexes–An in vitro and in vivo approach. Spectrochim. Acta Part A.

[90] Yamashita M., Watanabe Y., Mitsudo T.-a., Takegami Y. (1978). The Reductive Deacylation of the Knoevenagel Condensates by the Use of the Tetracarbonylhydridoferrate(0) Anion. A New Synthetic Route of Ketones from Aldehydes. Bull. Chem. Soc. Jpn..

[91] Cheng B., Yi H., He C., Liu C., Lei A. (2014). Revealing the Ligand Effect on Copper(I) Disproportionation via Operando IR Spectra. Organometallics.

[92] Hussein M.A., Huynh V.T., Hommelsheim R., Koenigs R.M., Nguyen T.V. (2018). An efficient method for retro-Claisen-type C-C bond cleavage of diketones with tropylium catalyst. Chem. Commun..

[93] Desai N.C., Kotadiya G.M., Vaja D.V. (2018). Synthesis and biological evaluation of some novel quinoline based pyrimidine derivatives. Indian J. Chem. Sect. B Org. Chem. Incl. Med. Chem..

[94] Kudyakova Y.S., Bazhin D.N., Slepukhin P.A., Burgart Y.V., Saloutin V.I., Charushin V.N. (2017). Unexpected formation of diethyl 2-ethoxy-6-CF_3_-2H-pyran-3,5-dicarboxylate from the condensation of ethyl 4,4,4-trifluoroacetoacetate with CH(OEt)_3_. Tetrahedron Lett..

[95] Bazhin D.N., Kudyakova Y.S., Nemytova N.A., Burgart Y.V., Saloutin V.I. (2016). Detrifluoroacetylation of 4,4,4-trifluoro-3,3-dihydroxy-2-(hydroxyimino)butan-1-ones as a convenient synthetic strategy for acyl cyanides. J. Fluor. Chem..

[96] Yang D.M., Zhou Y.H., Xue N., Qu J.P. (2013). Synthesis of Trifluoromethyl Ketones via Tandem Claisen Condensation and Retro-Claisen C-C Bond-Cleavage Reaction. J. Org. Chem..

[97] Biswas S., Maiti S., Jana U. (2010). An Efficient Iron-Catalyzed Carbon-Carbon Single-Bond Cleavage via Retro-Claisen Condensation: A Mild and Convenient Approach to Synthesize a Variety of Esters or Ketones. Eur. J. Org. Chem..

[98] Siirola E., Frank A., Grogan G., Kroutil W. (2013). C-C Hydrolases for Biocatalysis. Adv. Synth. Catal..

[99] Xie F., Yan F.X., Chen M.M., Zhang M. (2014). Base-catalyzed retro-Claisen condensation: A convenient esterification of alcohols via C-C bond cleavage of ketones to afford acylating sources. Rsc Adv..

[100] Jukic M., Sterk D., Casar Z. (2012). Recent Advances in the Retro-Claisen Reaction and Its Synthetic Applications. Curr. Org. Synth..

[101] Rao C.B., Rao D.C., Babu D.C., Venkateswarlu Y. (2010). Retro-Claisen Condensation with Fe-III as Catalyst under Solvent-Free Conditions. Eur. J. Org. Chem..

[102] Kawata A., Takata K., Kuninobu Y., Takai K. (2007). Indium-catalyzed retro-claisen condensation. Angew. Chem. Int. Ed. Engl..

[103] Johnson C.K. (1976). ORTEP, Report ORNL–5138.

[104] New and Improved CSD–The 2023 November Data Update (2023). Cambridge Structural Database (CSD). https://www.ccdc.cam.ac.uk/discover/blog/new-and-improved-csd-the-2023-november-data-update/.

[105] Allen F.H. (2002). The Cambridge Structural Database: A quarter of a million crystal structures and rising. Acta Crystallogr. Sect. B Struct. Sci..

[106] Volov A.N., Zamilatskov I.A. (2013). Ethyl N-(2-acetyl-3-oxo-1-phenylbutyl)carbamate. Acta Crystallogr. Sect. E Crystallogr. Commun..

[107] Gotsko M.D., Saliy I.V., Vashchenko A.V., Trofimov B.A. (2022). 1-Phenyl-3,3-di(1H-pyrazol-1-yl)propan-1-one. Molbank.

[108] Peters L., Hübner E., Haas T., Heinemann F.W., Burzlaff N. (2009). 3,3-Bis(3,5-dimethylpyrazol-1-yl)propionic acid: A tripodal N,N,O ligand for manganese and rhenium complexes*-*Syntheses and structures. J. Organomet. Chem..

[109] Kunz P.C., Berghahn M., Brückmann N.E., Dickmeis M., Kettel M., Spingler B. (2009). Functionalised Tris(pyrazolyl)methane Ligands and Re(CO)_3_ Complexes Thereof. Z. Anorg. Allg. Chem..

[110] Macrae C.F., Bruno I.J., Chisholm J.A., Edgington P.R., McCabe P., Pidcock E., Rodriguez-Monge L., Taylor R., van de Streek J., Wood P.A. (2008). Mercury CSD 2.0*-*New features for the visualization and investigation of crystal structures. J. Appl. Crystallogr..

[111] Malecki J.G. (2016). Personal communication.

[112] Okuniewski A., Rosiak D., Chojnacki J., Becker B. (2015). Coordination polymers and molecular structures among complexes of mercury(II) halides with selected 1-benzoylthioureas. Polyhedron.

[113] Yang L., Powell D.R., Houser R.P. (2007). Structural variation in copper(I) complexes with pyridylmethylamide ligands: Structural analysis with a new four-coordinate geometry index, τ4. Dalton Trans..

[114] (2022). CrysAlisPro. Versions 1.171.41.123a and 1.171.42.49 (Rigaku Oxford Diffraction).

[115] Sheldrick G.M. (2015). SHELXT*-*Integrated space-group and crystal-structure determination. Acta Crystallogr. Sect. A Found. Adv..

[116] Sheldrick G.M. (2015). Crystal structure refinement with SHELXL. Acta Crystallogr. Sect. C Struct. Chem..

[117] Dolomanov O.V., Bourhis L.J., Gildea R.J., Howard J.A.K., Puschmann H. (2009). OLEX2: A complete structure solution, refinement and analysis program. J. Appl. Crystallogr..

