# Supplementary material for: New Copper Complexes with N,O-Donor Ligands Based on Pyrazole Moieties Supported by 3-Substituted Acetylacetone Scaffolds
Source: Molecules. 2024 Jan 28;29(3):621. doi: 10.3390/molecules29030621 (PMC10856362; doi:10.3390/molecules29030621)
Supplement: Supplementary file 1 [file molecules-29-00621-s001.zip › molecules-2827305-supplementary.pdf]

Supporting Information

New copper complexes with N,O-donor ligands based on pyrazole moieties supported by 3-substituted acetylacetone scaffold

Jo' Del Gobbo [1], Carlo Santini [1], Alessandro Dolmella [2], ZhenZhen Li [1], Miriam Caviglia [1], Maura Pellei [1]

[1] School of Science and Technology, Chemistry Division, University of Camerino, Via Madonna delle Carceri (ChIP), Camerino, 62032 Macerata, Italy; jo.delgobbo@unicam.it (J.D.G.); carlo.santini@unicam.it (C.S.); zhenzhen.li@unicam.it (Z.L.); miriam.caviglia@unicam.it (M.C.)

[2] Department of Pharmaceutical and Pharmacological Sciences, University of Padova, Via Marzolo 5, 35131 Padova, Italy

* Correspondence: alessandro.dolmella@unipd.it (A.D.); maura.pellei@unicam.it (M.P.)

Table of Contents:

Figure S1. A crystal packing representation of compound **1** viewed down the crystallographic *b* axis, showing the shortest intermolecular contacts described in Table 3. Only the atoms involved in the contacts are labelled. Distances and angles of each contact are highlighted along the pertinent interaction.

Figure S2. A crystal packing representation of compound **2** viewed down the crystallographic *c* axis, showing the shortest intermolecular contacts described in Table 3. Only the atoms involved in the contacts are labelled. Distances and angles of each contact are highlighted along the pertinent interaction.

Figure S3. A crystal packing representation of compound **2** viewed down the crystallographic *b* axis, showing in particular the O2....H16 close contact that pairs two proximal chains propagating along the crystallographic *a* axis. Distances and angles of the contact are highlighted along the interaction.

Figure S4. A crystal packing representation of compound **8** viewed down the crystallographic *b* axis, showing the shortest intermolecular contacts described in Table 3. Only the atoms involved in the contacts are labelled. Distances and angles of each contact are highlighted along the pertinent interaction.

Figure S5. A crystal packing representation of compound **3** viewed down the crystallographic *b* axis, showing the only intermolecular contact not depending on the PF_6^- anion described in Table 3, i.e., the C15....H37 contact that defines a linear chain motif propagating along the crystallographic *a* axis. Only the atoms involved in the contacts are labelled. Distance and angle of the contact highlighted along the interaction.

Figure S6. A crystal packing representation of compound **3** viewed down the crystallographic *b* axis, showing the alternate layers containing the cationic complex and the hexafluorophosphate anion piled up along the crystallographic *c* axis.

Figure S7. A crystal packing representation of compound **3** viewed down the crystallographic *a* axis, showing the zigzag chain motif developing along the crystallographic *c* axis that involves the F2, F2A, F5A and F6A positions occupied by the hexafluorophosphate anion. Only the atoms involved in the contacts are labelled. Distances and angles of the contacts highlighted along the interactions.

Figure S8. A crystal packing representation of compound **3** viewed down the crystallographic *b* axis, showing another chain motif which runs along the -1, 1, 0 plane involving the F1A, F3, F4 and F4A positions occupied by the hexafluorophosphate anion that criss-crosses with the zigzag motif running along the crystallographic *c* axis as well with the C15....H37 contact (also these atoms labelled). Distances and angles of the contacts highlighted along the interactions.

Figure S9. FT-IR spectrum of HL^{acPz} (**1**).

Figure S10. ^1H-NMR spectrum of HL^{acPz} (**1**) in $CDCl_3$.

Figure S11. ^1H-NMR spectrum of HL^{acPz} (**1**) in DMSO-d_6.

Figure S12. ^1H-NMR spectrum of HL^{acPz} (**1**) in Acetone-d_6.

Figure S13. $^{13}C\{^1H\}$-NMR spectrum of HL^{acPz} (**1**) in $CDCl_3$.

Figure S14. ^{13}C{^{1}H}-NMR spectrum of HL^{acPz} (**1**) in Acetone-d$_6$.
Figure S15. FT-IR spectrum of HL^{acPzMe} (**2**).
Figure S16. ^{1}H-NMR spectrum of HL^{acPzMe} (**2**) in $CDCl_3$.
Figure S17. ^{13}C{^{1}H}-NMR spectrum of HL^{acPzMe} (**2**) in $CDCl_3$.
Figure S18. FT-IR spectrum of [Cu(HL^{acPz})(PPh_3)$_2$]PF_6 (**3**).
Figure S19. ^{1}H-NMR spectrum of [Cu(HL^{acPz})(PPh_3)$_2$]PF_6 (**3**) in Acetone-d$_6$.
Figure S20. ^{13}C{^{1}H}-NMR spectrum of [Cu(HL^{acPz})(PPh_3)$_2$]PF_6 (**3**) in Acetone-d$_6$.
Figure S21. ^{31}P{^{1}H}-NMR spectrum of [Cu(HL^{acPz})(PPh_3)$_2$]PF_6 (**3**) in $CDCl_3$.
Figure S22. FT-IR spectrum of [Cu(HL^{acPz})$_2$(L^{acPz})$_2$] (**4**).
Figure S23. FT-IR spectrum of [Cu(HL^{acPzMe})(PPh_3)$_2$]PF_6·2CH_3CN (**5**).
Figure S24. ^{1}H-NMR spectrum of [Cu(HL^{acPzMe})(PPh_3)$_2$]PF_6·2CH_3CN (**5**) in Acetone-d$_6$.
Figure S25. ^{13}C{^{1}H}-NMR spectrum of [Cu(HL^{acPzMe})(PPh_3)$_2$]PF_6·2CH_3CN (**5**) in Acetone-d$_6$.
Figure S26. ^{31}P{^{1}H}-NMR spectrum of [Cu(HL^{acPzMe})(PPh_3)$_2$]PF_6·2CH_3CN (**5**) in $CDCl_3$.
Figure S27. FT-IR spectrum of [Cu(HL^{acPzMe})$_2$(L^{acPzMe})$_2$] (**6**).
Figure S28. FT-IR spectrum of ^{PhPz}MEK (**7**).
Figure S29. ^{1}H-NMR spectrum of ^{PhPz}MEK (**7**) in $CDCl_3$.
Figure S30. ^{13}C{^{1}H}-NMR spectrum of ^{PhPz}MEK (**7**) in $CDCl_3$.
Figure S31. FT-IR spectrum of ^{PhPzMe2}MEK (**8**).
Figure S32. ^{1}H-NMR spectrum of ^{PhPzMe2}MEK (**8**) in $CDCl_3$.
Figure S33. ^{13}C{^{1}H}-NMR spectrum of ^{PhPzMe2}MEK (**8**) in $CDCl_3$.

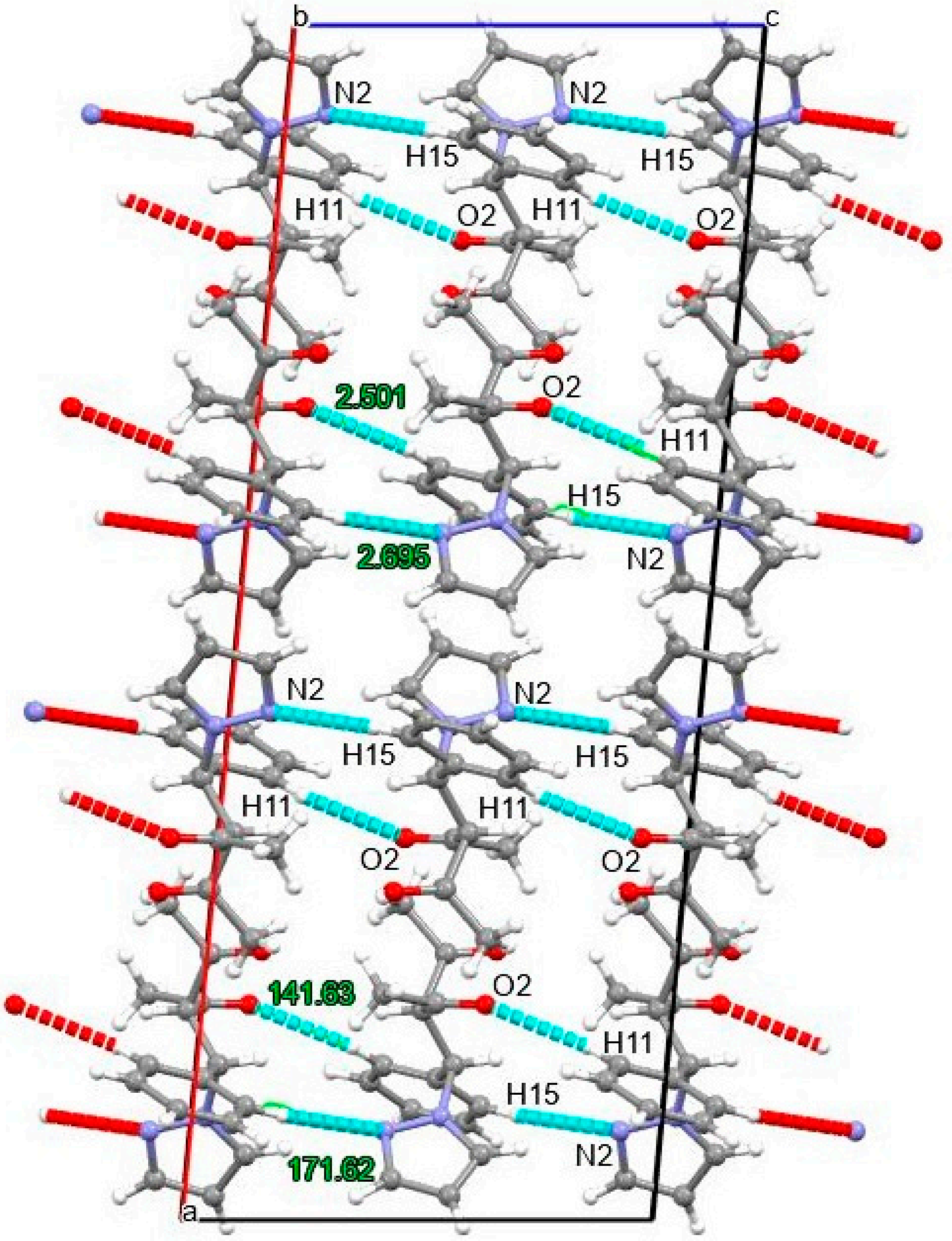

Figure S1. A crystal packing representation of compound **1** viewed down the crystallographic *b* axis, showing the shortest intermolecular contacts described in Table 3. Only the atoms involved in the contacts are labelled. Distances and angles of each contact are highlighted along the pertinent interaction.

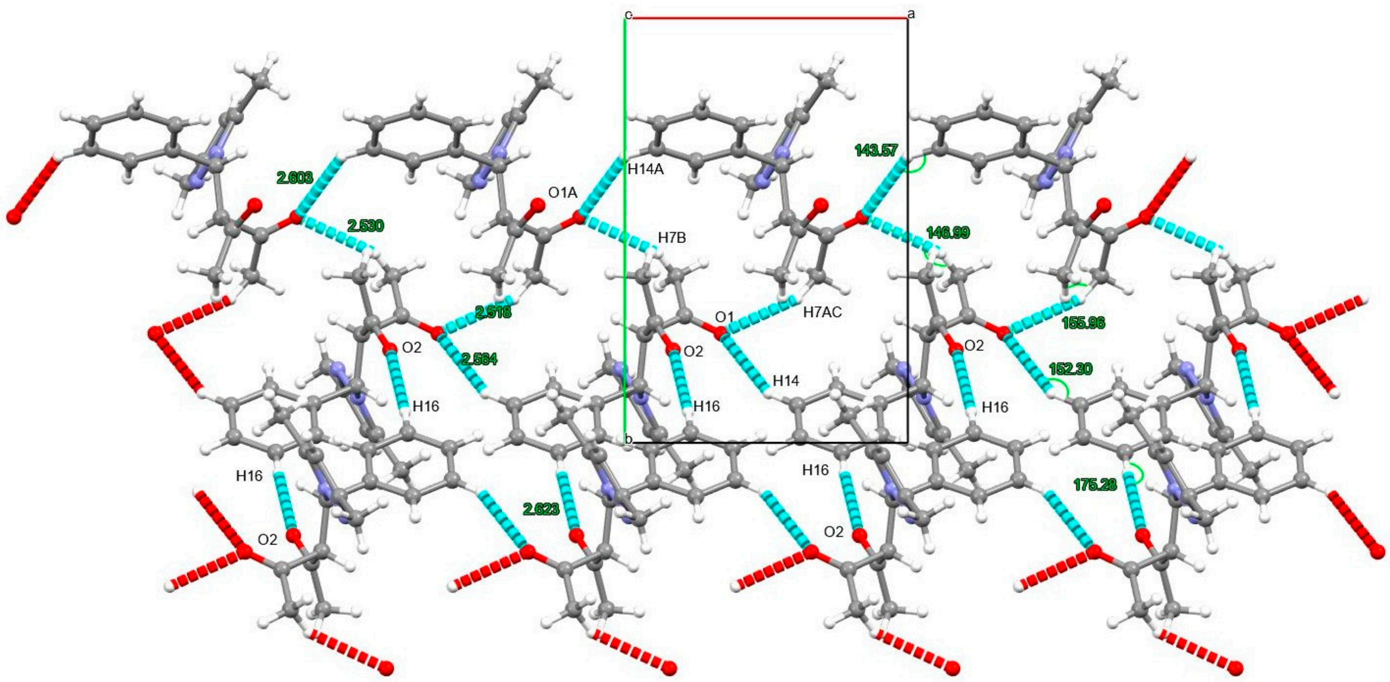

Figure S2. A crystal packing representation of compound **2** viewed down the crystallographic *c* axis, showing the shortest intermolecular contacts described in Table 3. Only the atoms involved in the contacts are labelled. Distances and angles of each contact are highlighted along the pertinent interaction.

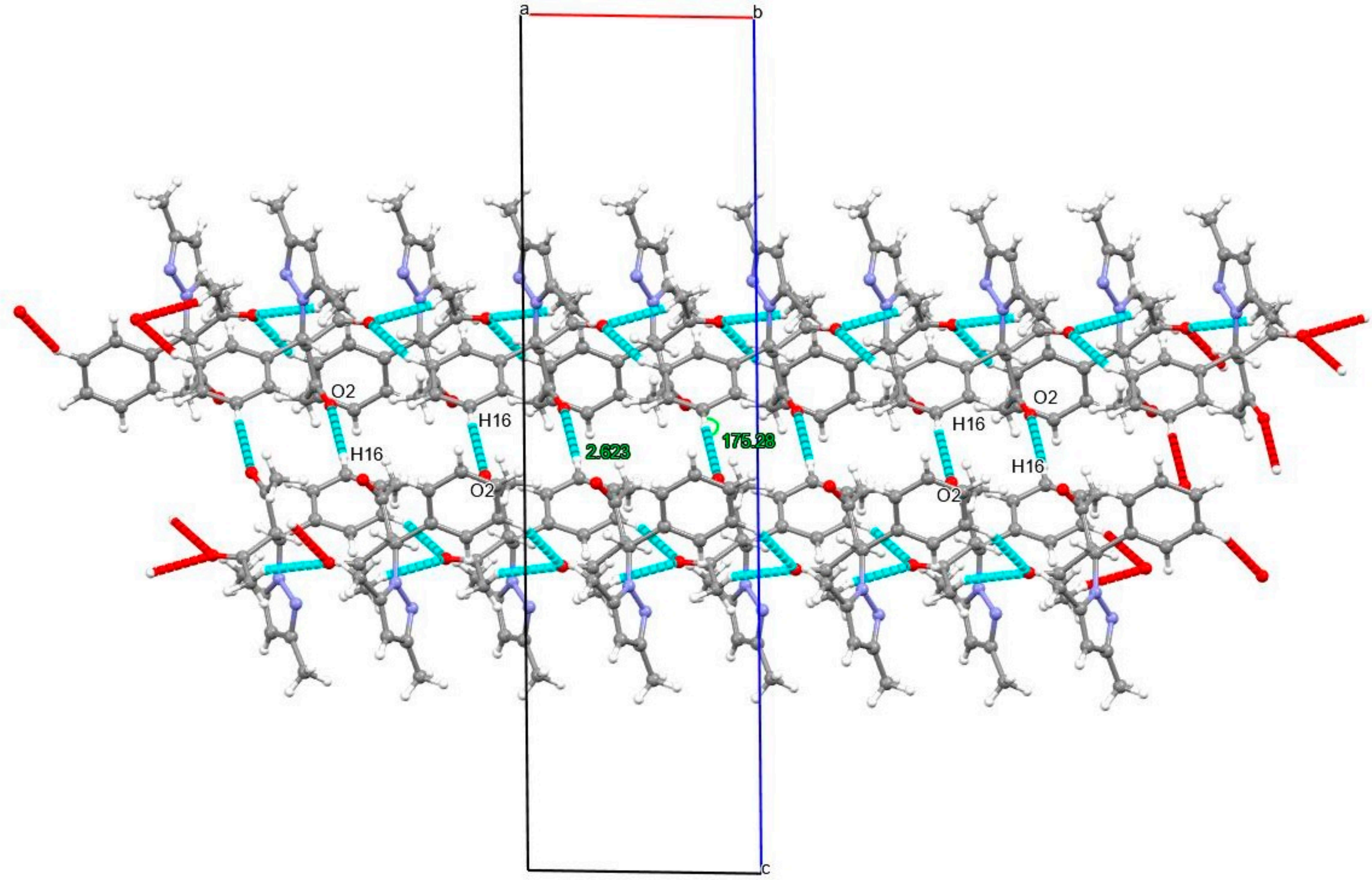

Figure S3. A crystal packing representation of compound **2** viewed down the crystallographic *b* axis, showing in particular the O2....H16 close contact that pairs two proximal chains propagating along the crystallographic *a* axis. Distances and angles of the contact are highlighted along the interaction.

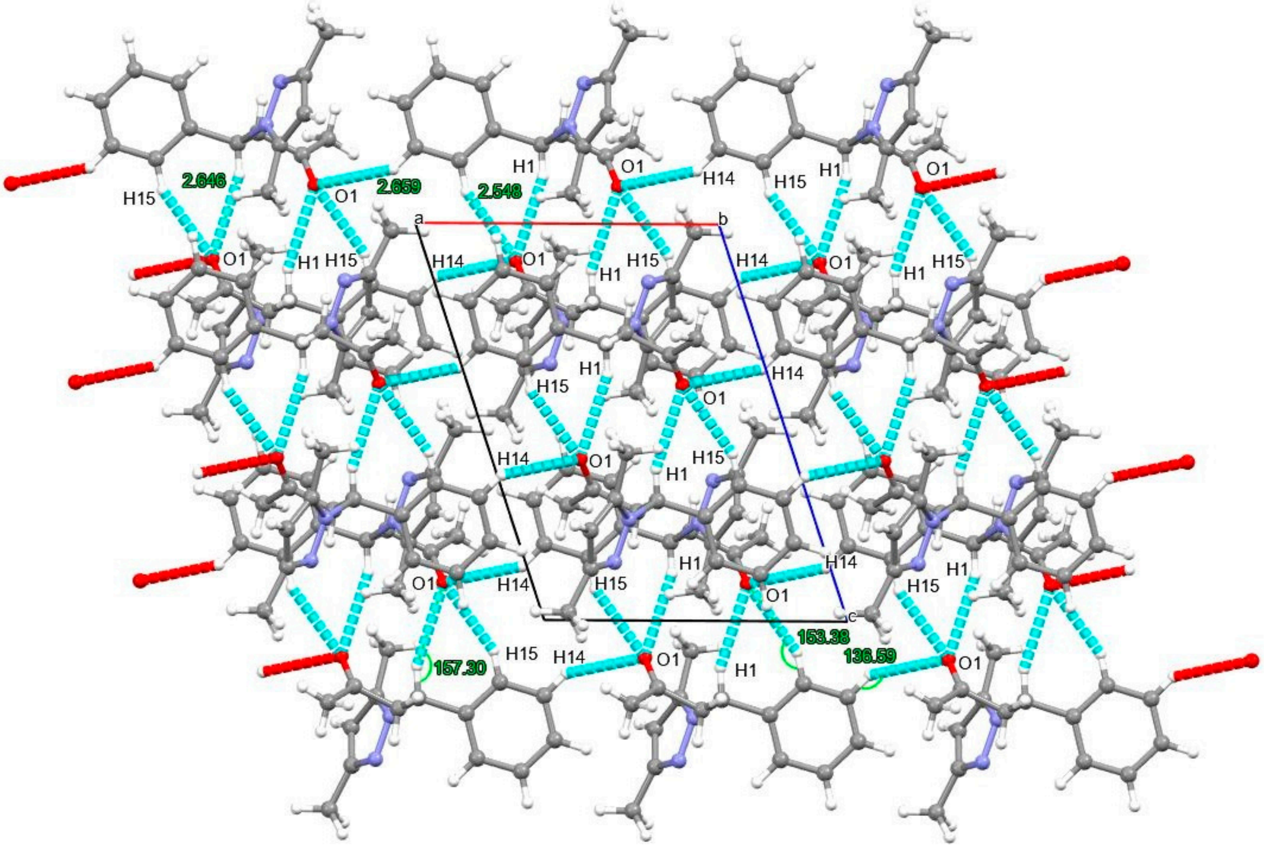

Figure S4. A crystal packing representation of compound **8** viewed down the crystallographic *b* axis, showing the shortest intermolecular contacts described in Table 3. Only the atoms involved in the contacts are labelled. Distances and angles of each contact are highlighted along the pertinent interaction.

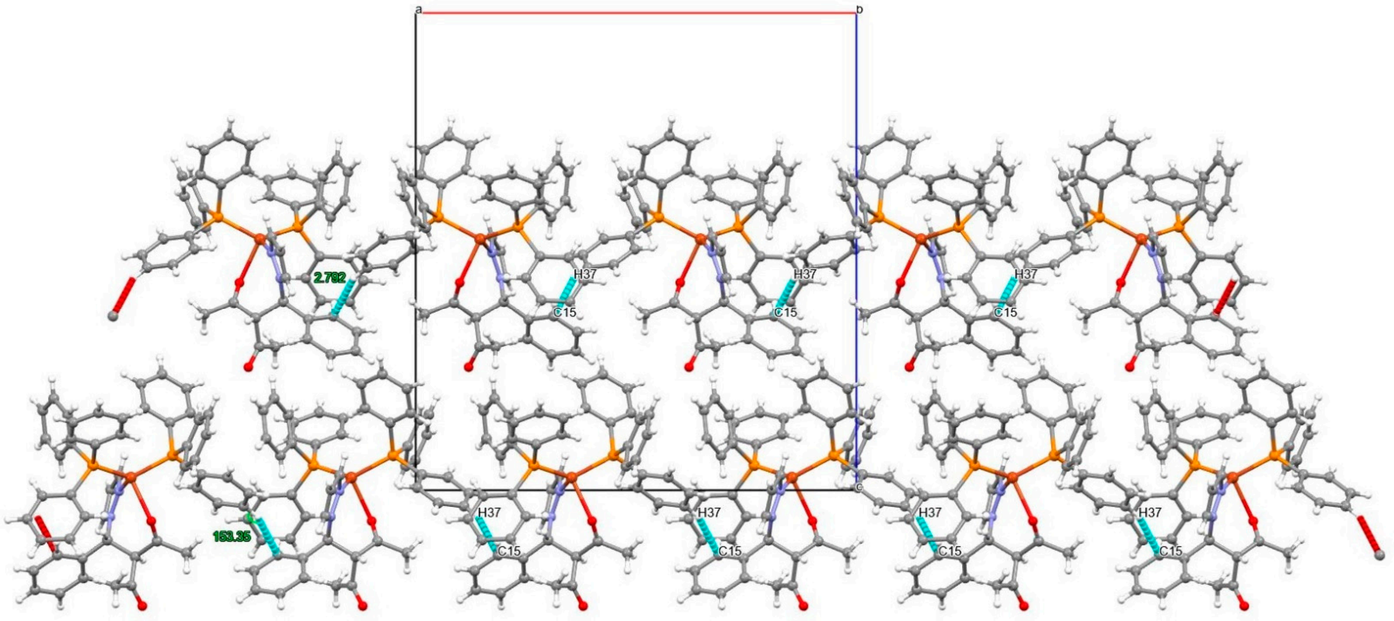

Figure S5. A crystal packing representation of compound **3** viewed down the crystallographic *b* axis, showing the only intermolecular contact not depending on the PF_6^- anion described in Table 3, i.e., the C15....H37 contact that defines a linear chain motif propagating along the crystallographic *a* axis. Only the atoms involved in the contacts are labelled. Distance and angle of the contact highlighted along the interaction.

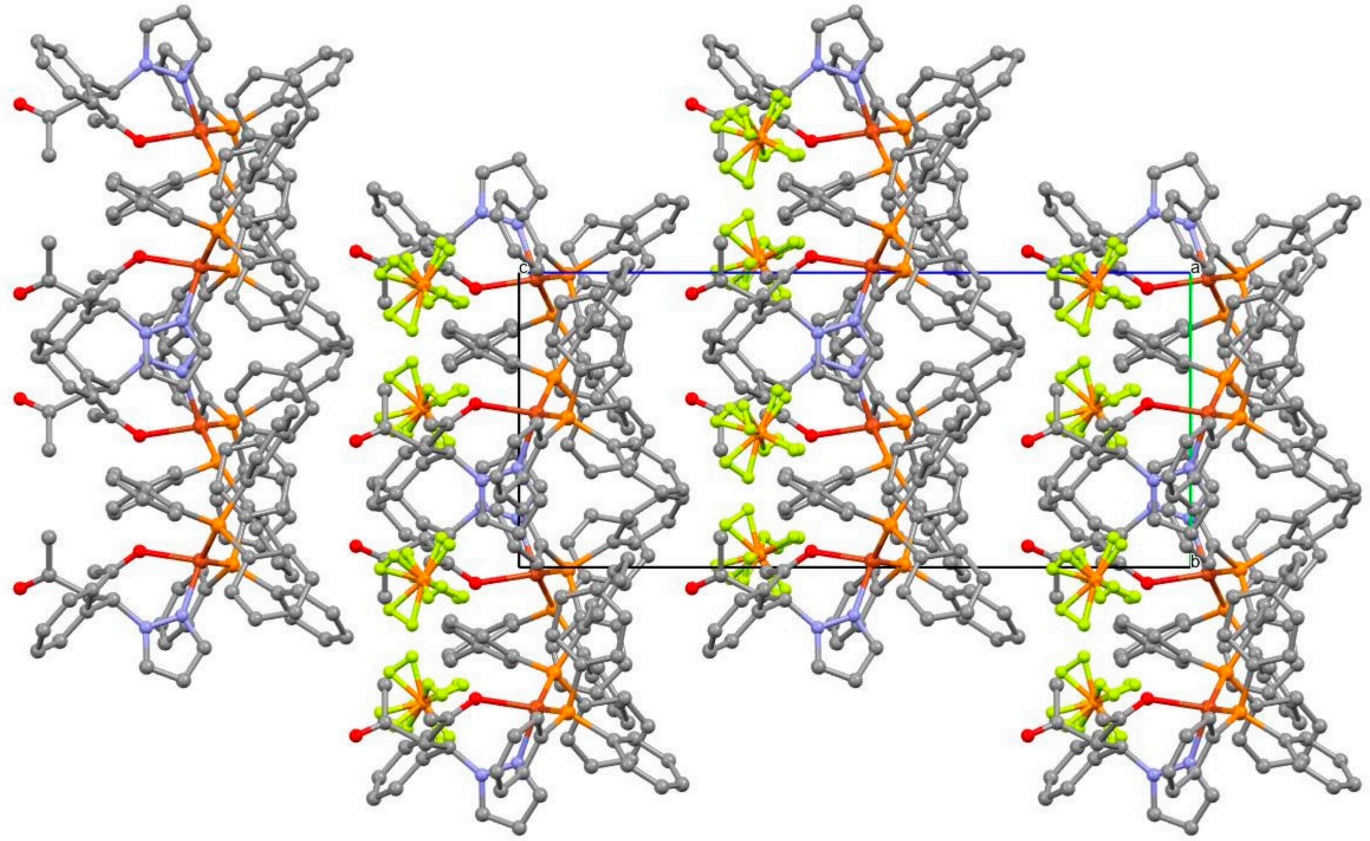

Figure S6. A crystal packing representation of compound **3** viewed down the crystallographic *b* axis, showing the alternate layers containing the cationic complex and the hexafluorophosphate anion piled up along the crystallographic *c* axis.

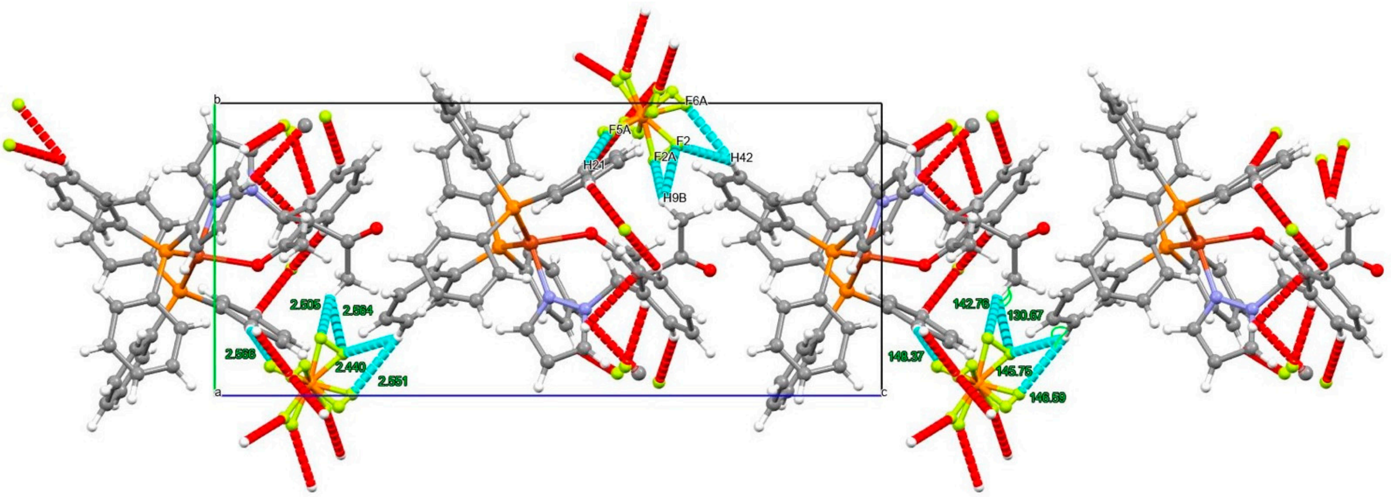

Figure S7. A crystal packing representation of compound **3** viewed down the crystallographic *a* axis, showing the zigzag chain motif developing along the crystallographic *c* axis that involves the F2, F2A, F5A and F6A positions occupied by the hexafluorophosphate anion. Only the atoms involved in the contacts are labelled. Distances and angles of the contacts highlighted along the interactions.

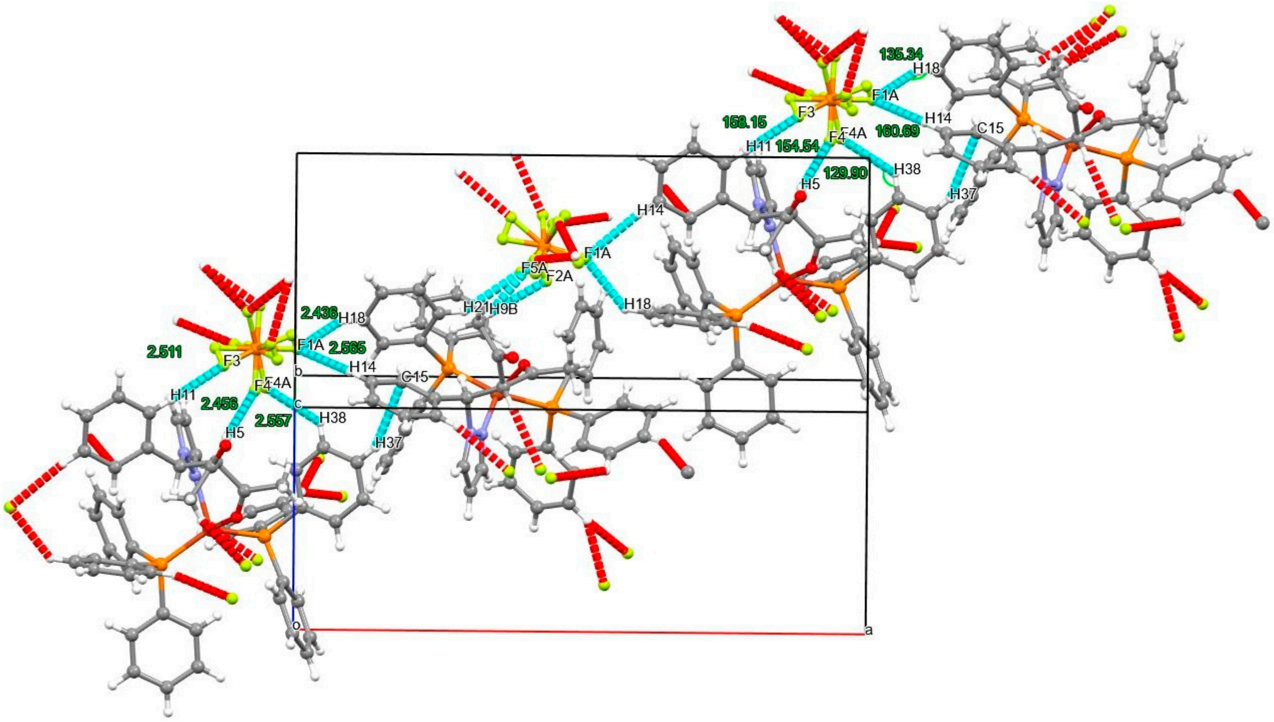

Figure S8. A crystal packing representation of compound **3** viewed down the crystallographic *b* axis, showing another chain motif which runs along the -1, 1, 0 plane involving the F1A, F3, F4 and F4A positions occupied by the hexafluorophosphate anion that criss-crosses with the zigzag motif running along the crystallographic *c* axis as well with the C15....H37 contact (also these atoms labelled). Distances and angles of the contacts highlighted along the interactions.

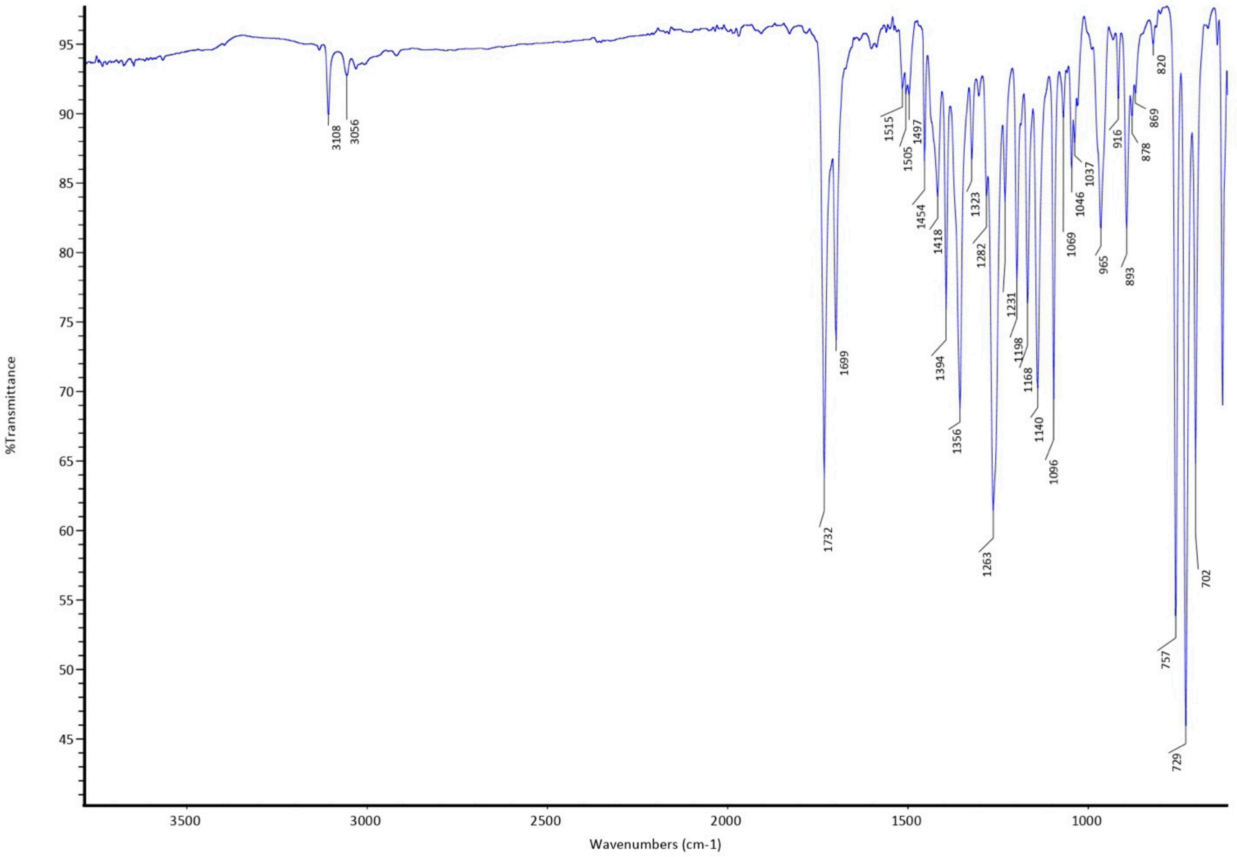

Figure S9. FT-IR spectrum of HL^{acPz} (**1**).

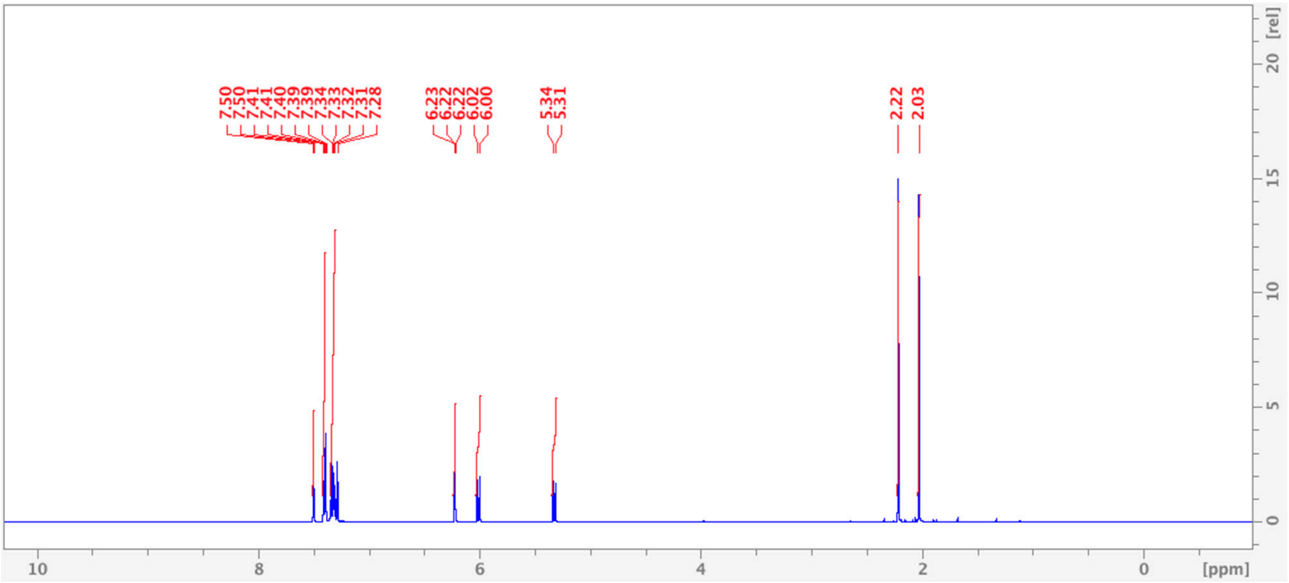

Figure S10. ^{1}H-NMR spectrum of HL^{acPz} (**1**) in $CDCl_3$.

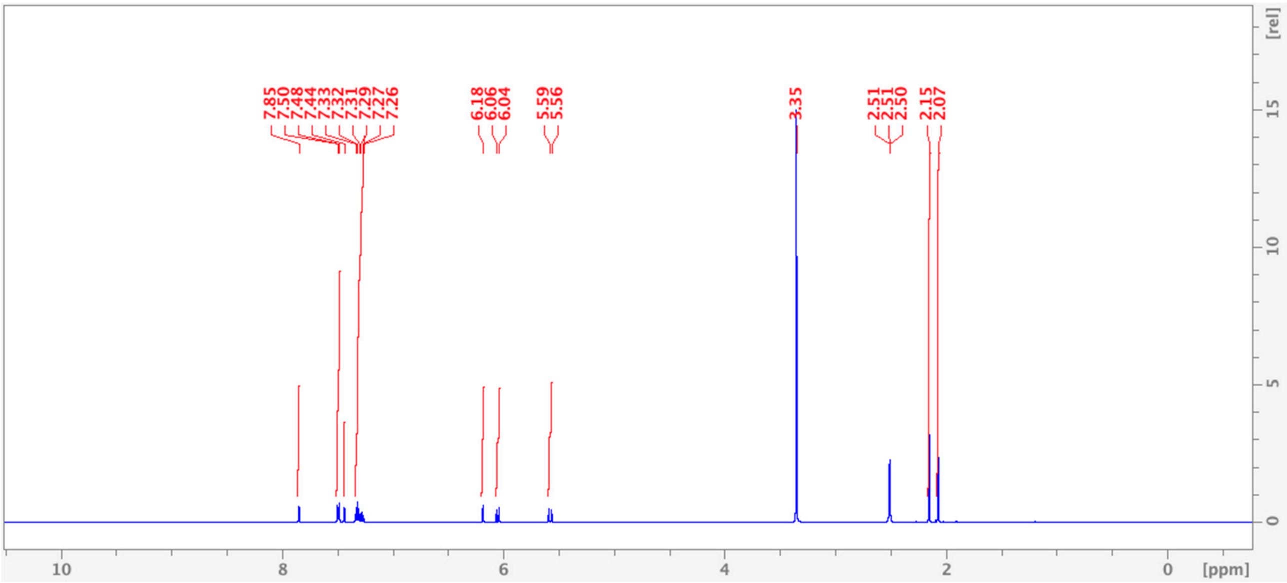

Figure S11. ^{1}H-NMR spectrum of HL^{acPz} (**1**) in DMSO-d_6.

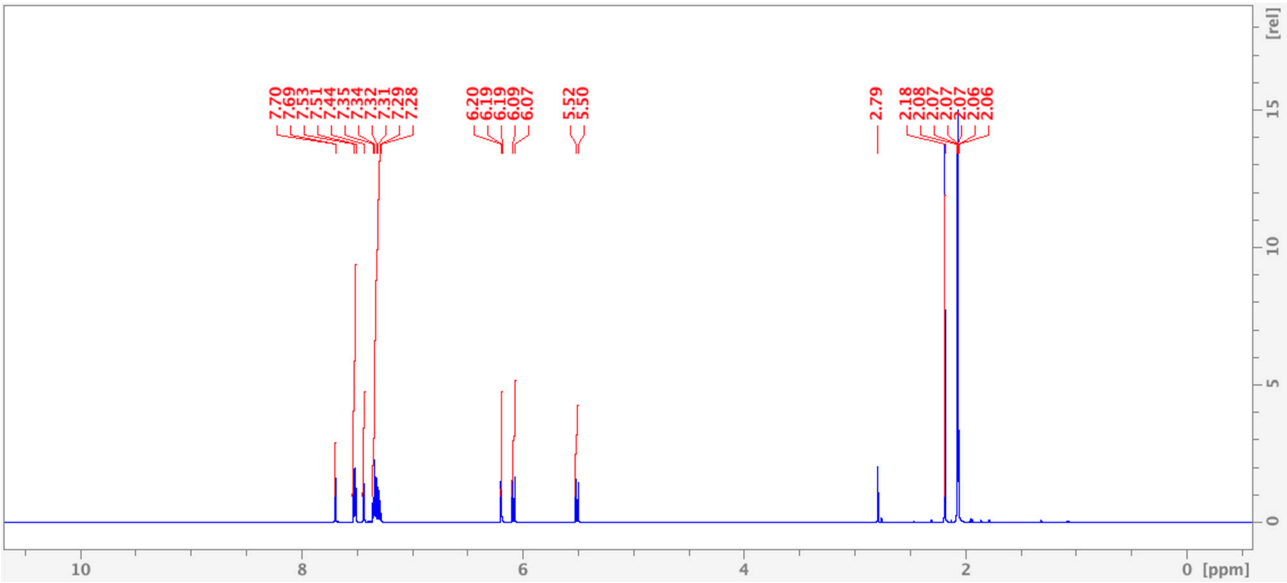

Figure S12. ^{1}H-NMR spectrum of HL^{acPz} (**1**) in Acetone-d_6.

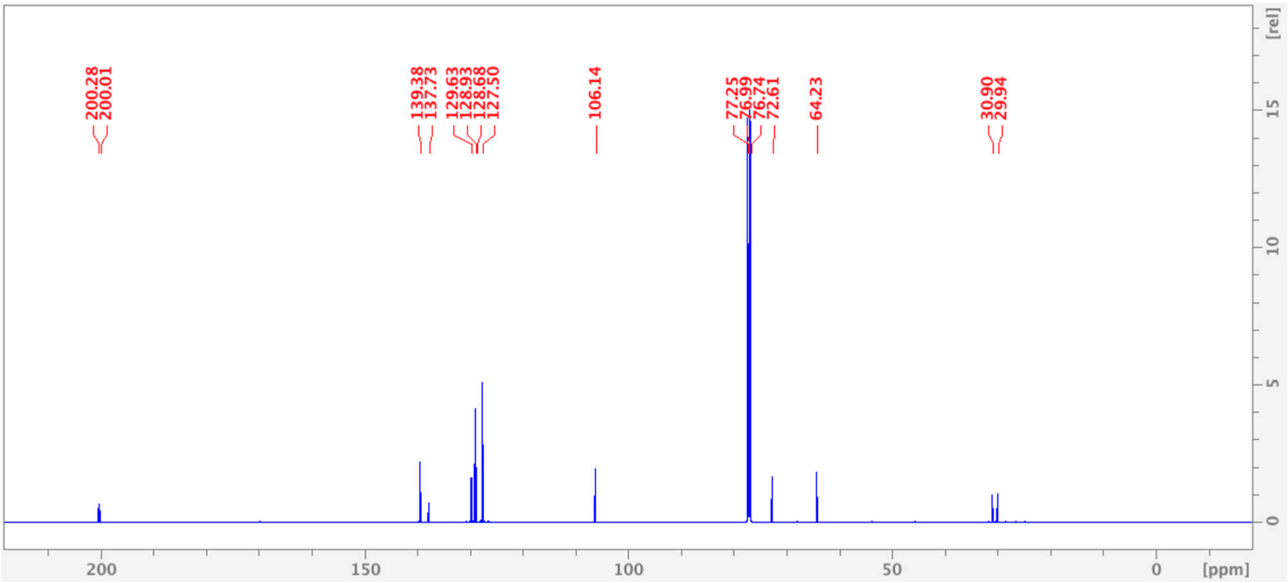

Figure S13. $^{13}C\{^{1}H\}$-NMR spectrum of HL^{acPz} (**1**) in $CDCl_3$.

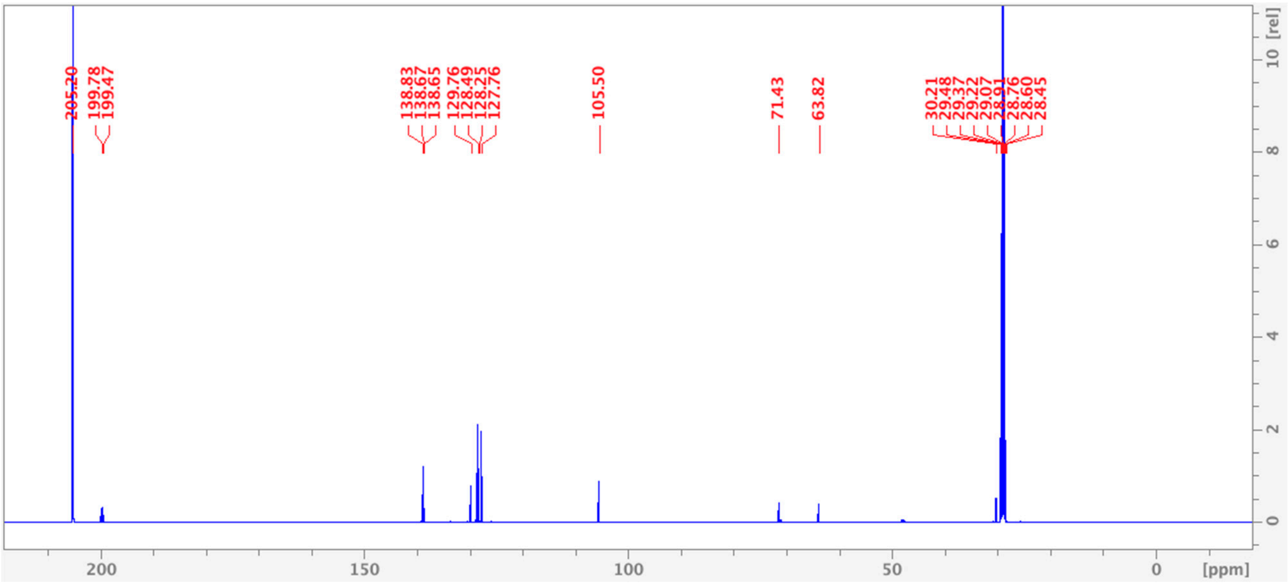

Figure S14. $^{13}C\{^{1}H\}$-NMR spectrum of HL^{acPz} (**1**) in Acetone-d_6.

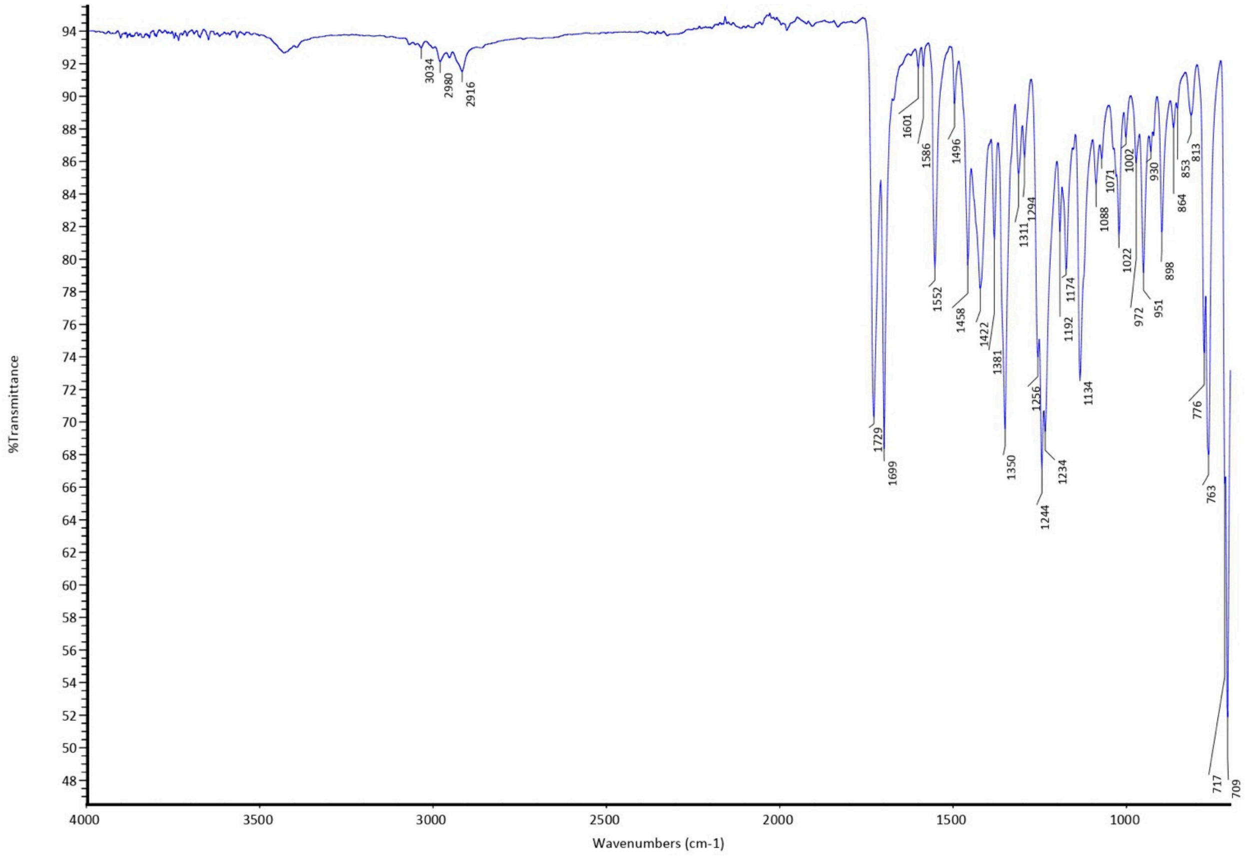

Figure S15. FT-IR spectrum of HL^{acPzMe} (**2**).

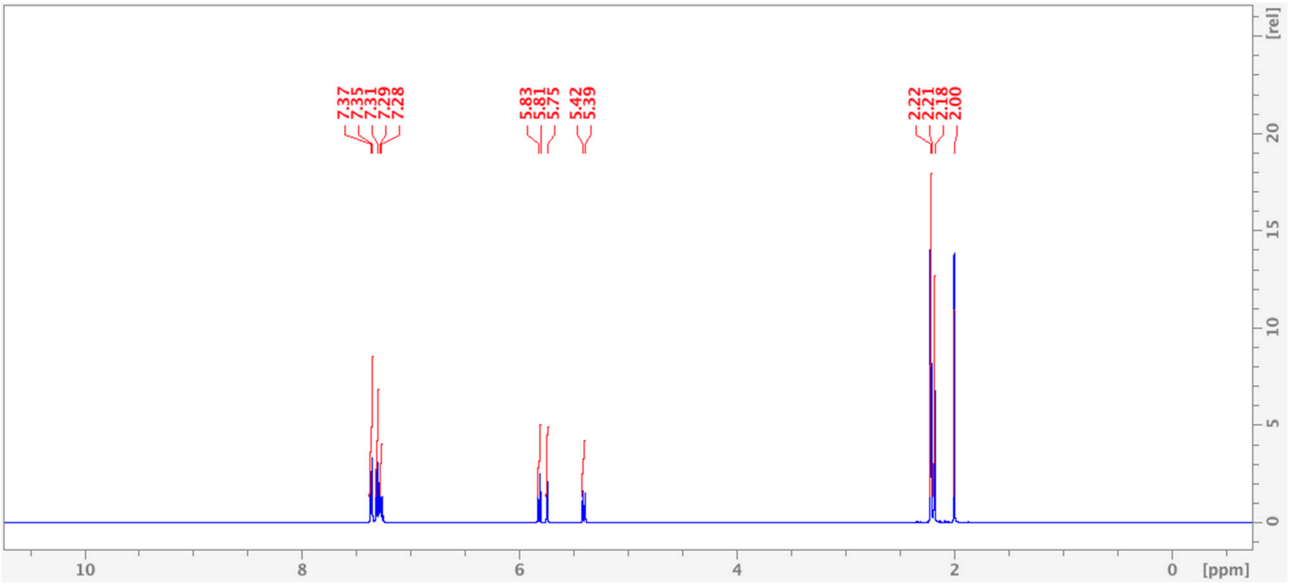

Figure S16. ^{1}H-NMR spectrum of HL^{acPzMe} (**2**) in $CDCl_3$.

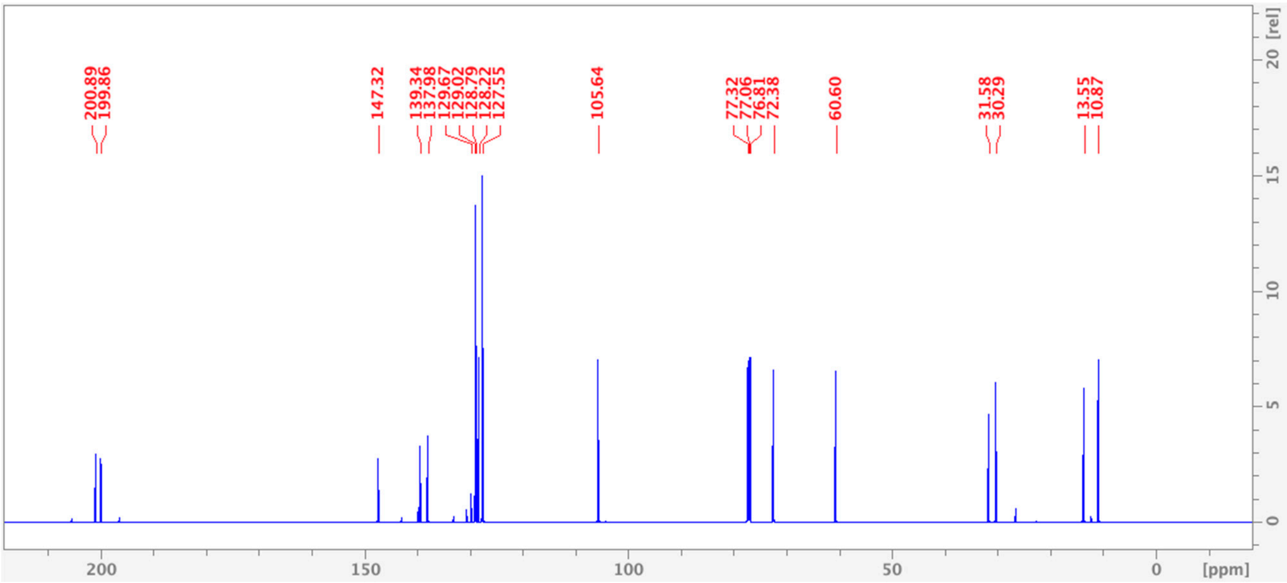

Figure S17. $^{13}C\{^{1}H\}$-NMR spectrum of HL^{acPzMe} (**2**) in $CDCl_3$.

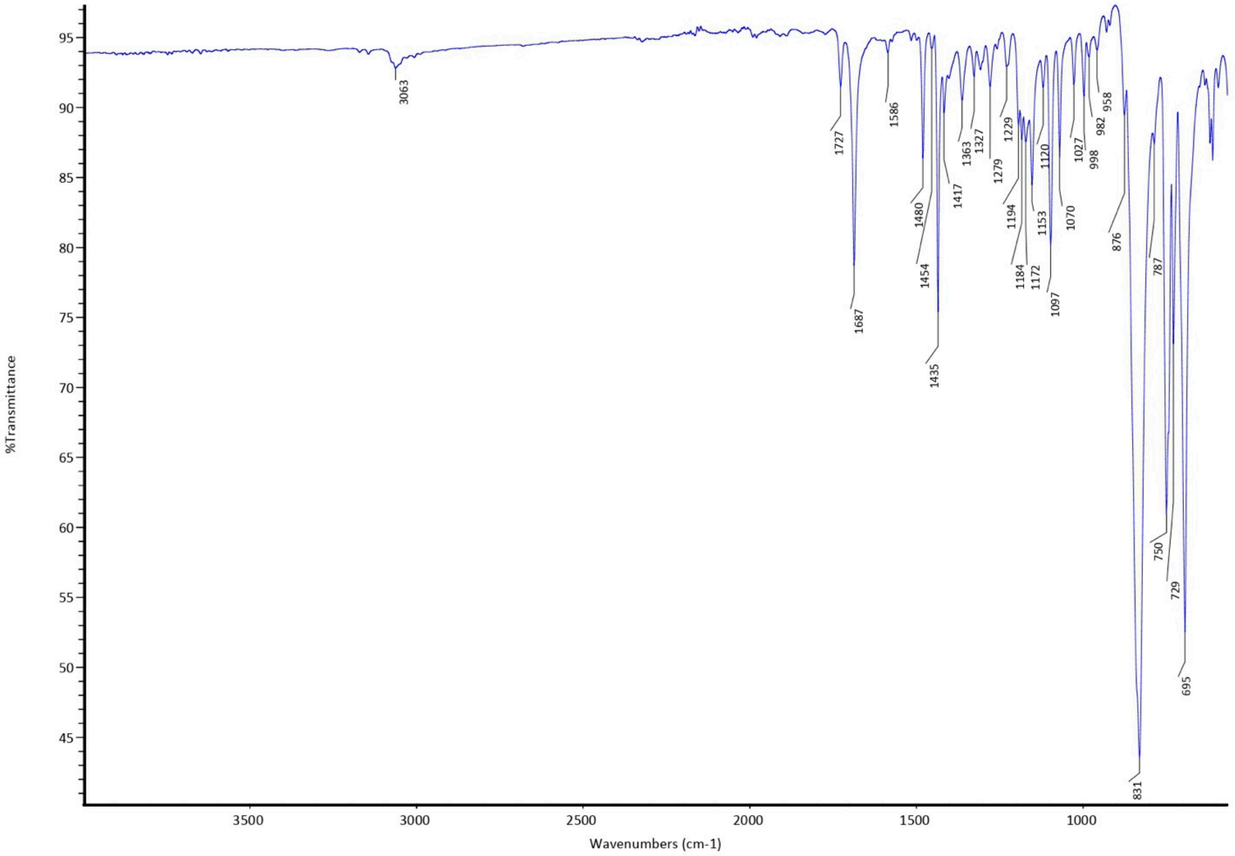

Figure S18. FT-IR spectrum of $[Cu(HL^{acPz})(PPh_3)_2]PF_6$ (**3**).

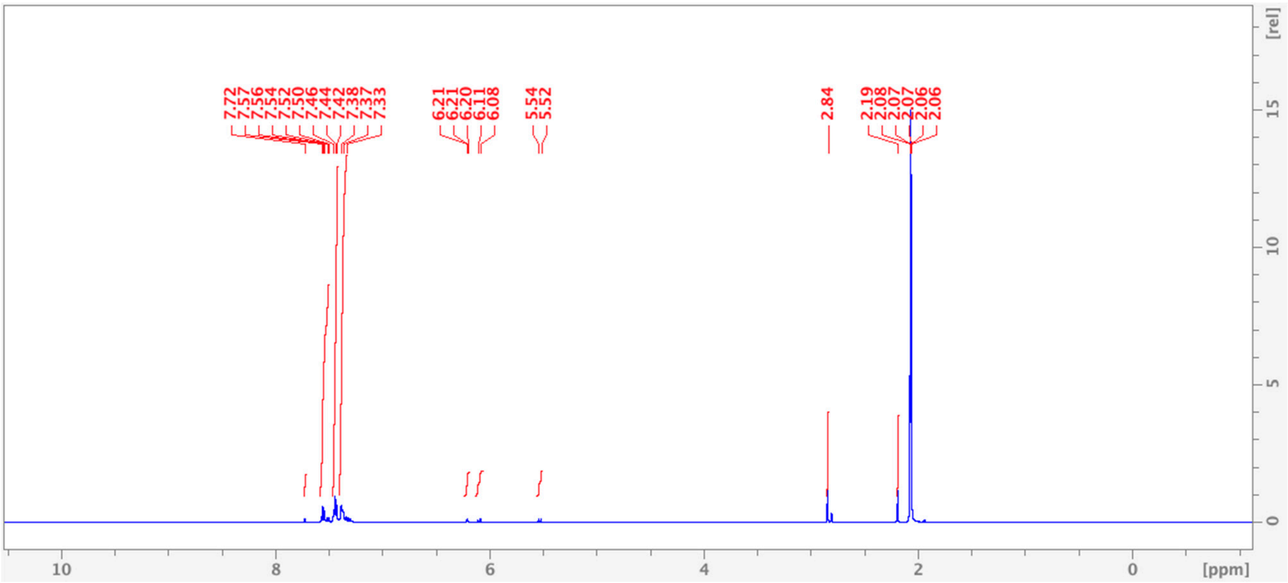

Figure S19. ^{1}H-NMR spectrum of $[Cu(HL^{acPz})(PPh_3)_2]PF_6$ (**3**) in Acetone-d_6.

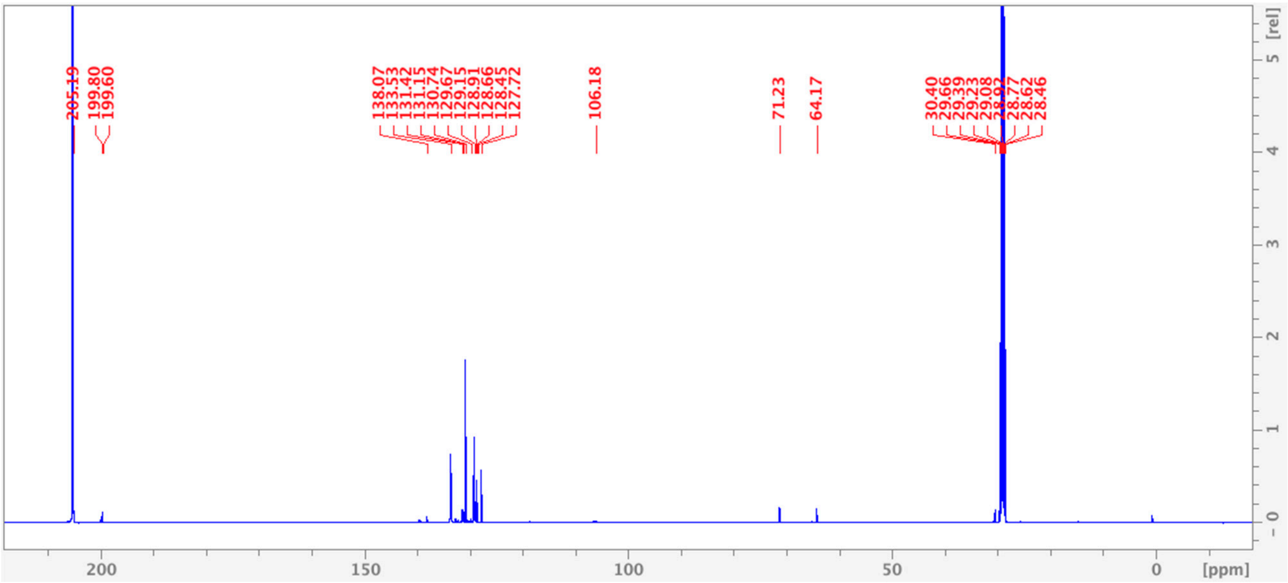

Figure S20. $^{13}C\{^{1}H\}$-NMR spectrum of $[Cu(HL^{acPz})(PPh_3)_2]PF_6$ (**3**) in Acetone-d_6.

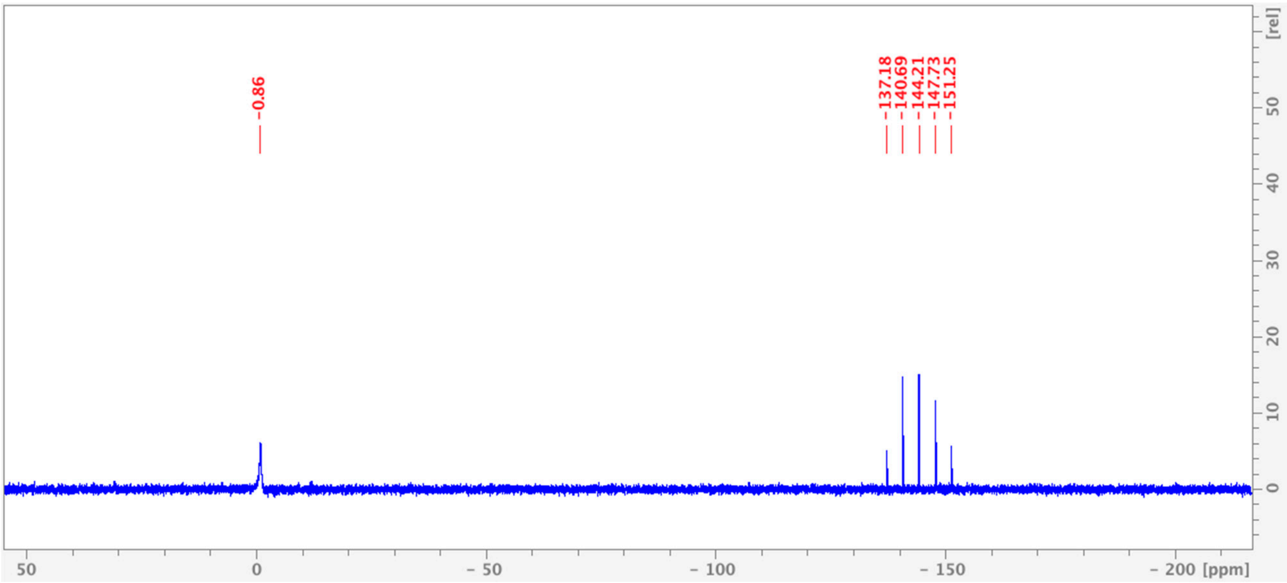

Figure S21. $^{31}P\{^{1}H\}$-NMR spectrum of $[Cu(HL^{acPz})(PPh_3)_2]PF_6$ (**3**) in $CDCl_3$.

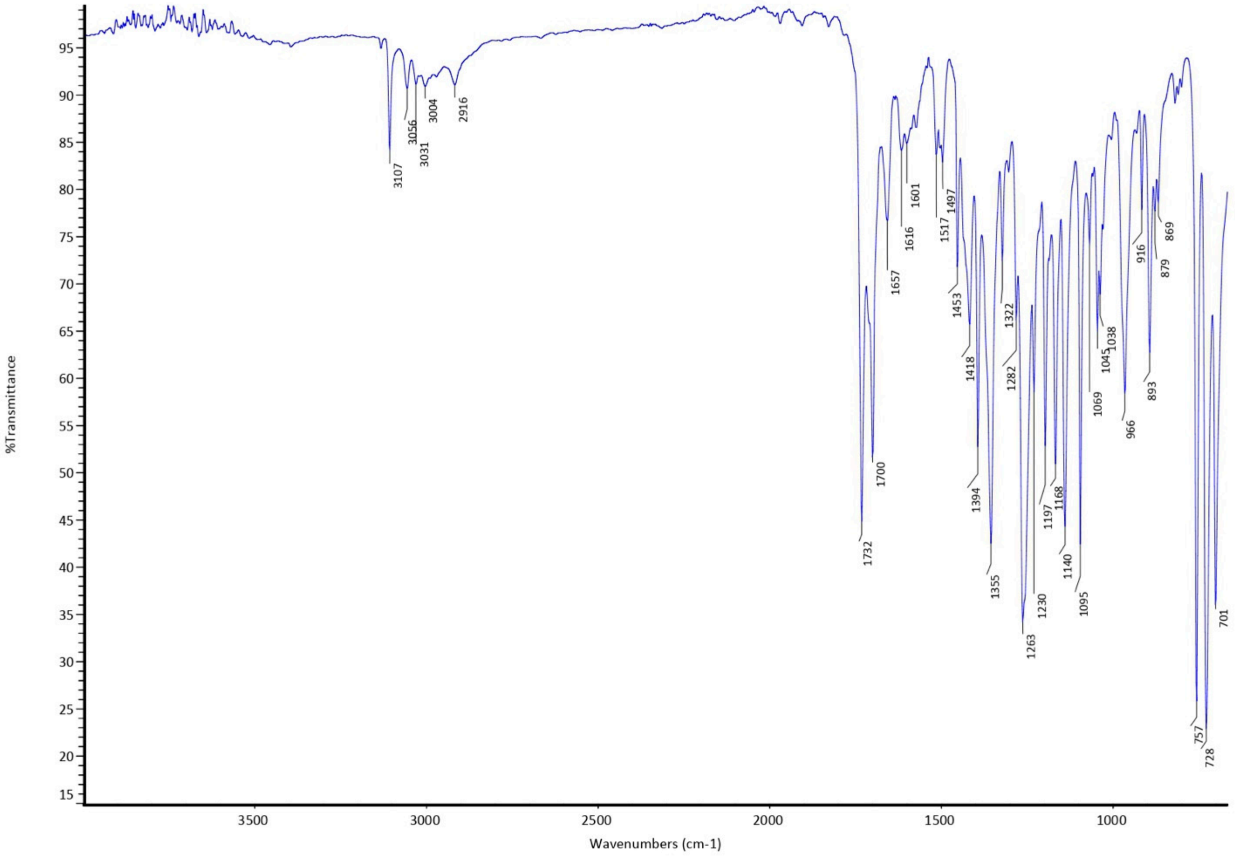

Figure S22. FT-IR spectrum of $[Cu(HL^{acPz})_2(L^{acPz})_2]$ (**4**).

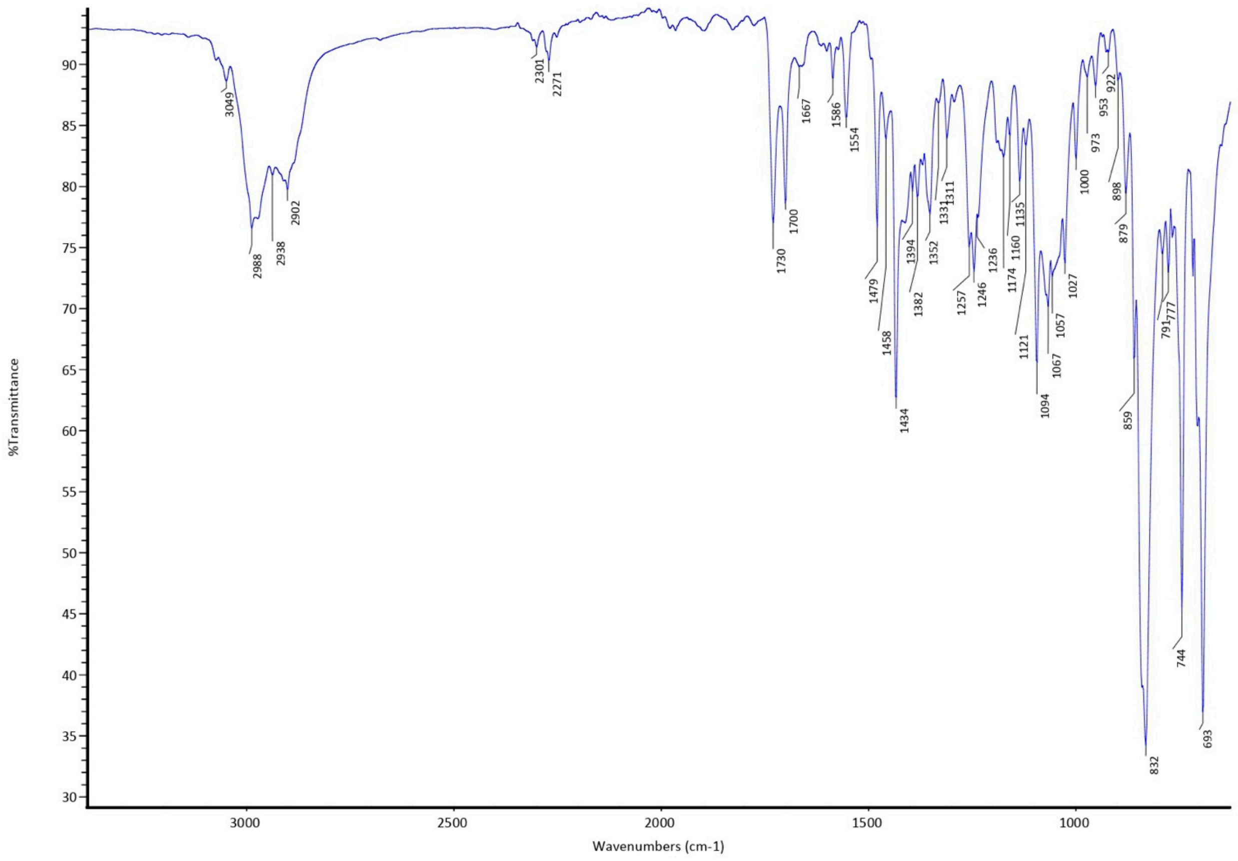

Figure S23. FT-IR spectrum of $[Cu(HL^{acPzMe})(PPh_3)_2]PF_6 \cdot 2CH_3CN$ (**5**).

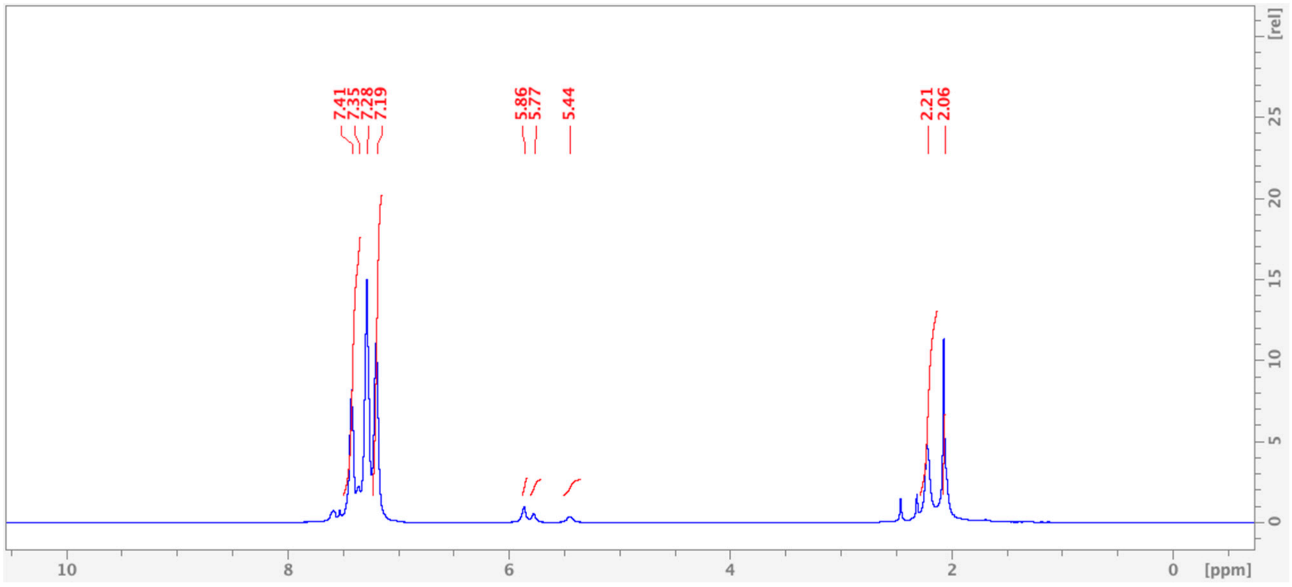

Figure S24. ^{1}H-NMR spectrum of $[Cu(HL^{acPzMe})(PPh_3)_2]PF_6 \cdot 2CH_3CN$ (**5**) in Acetone-d_6.

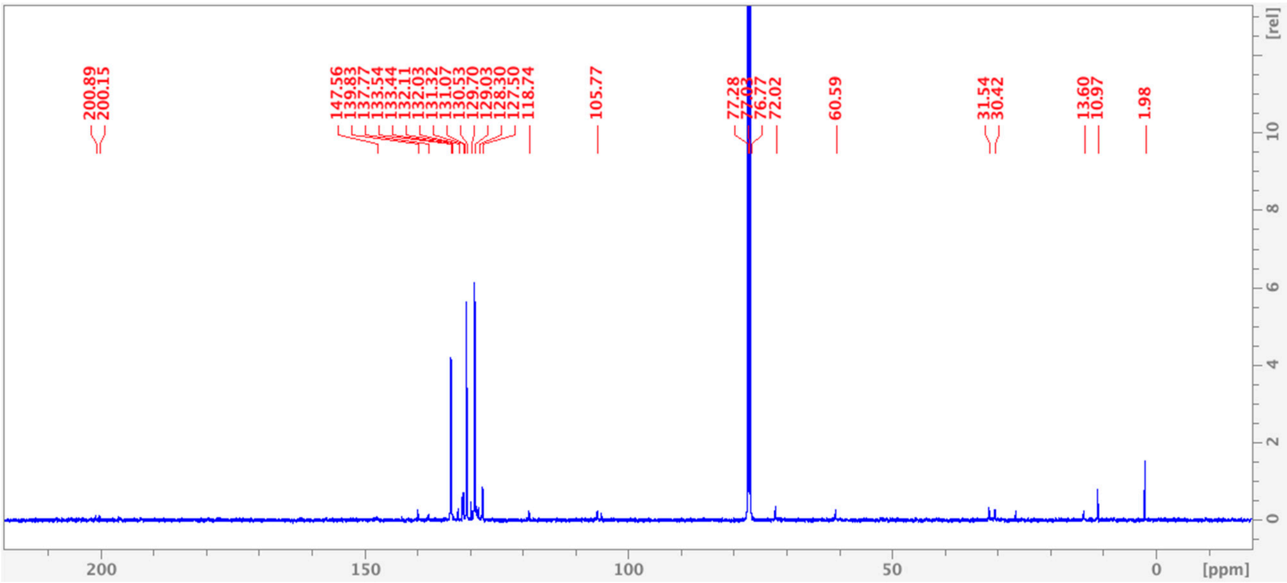

Figure S25. $^{13}C\{^1H\}$-NMR spectrum of $[Cu(HL^{acPzMe})(PPh_3)_2]PF_6 \cdot 2CH_3CN$ (**5**) in Acetone-d_6.

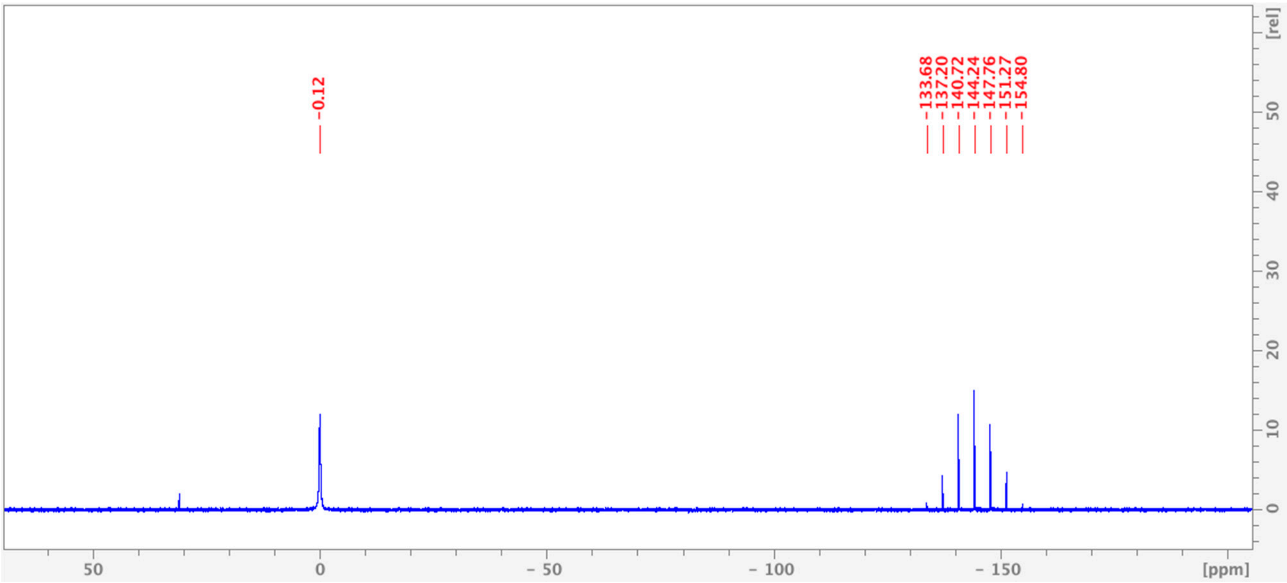

Figure S26. $^{31}P\{^1H\}$-NMR spectrum of $[Cu(HL^{acPzMe})(PPh_3)_2]PF_6 \cdot 2CH_3CN$ (**5**) in $CDCl_3$.

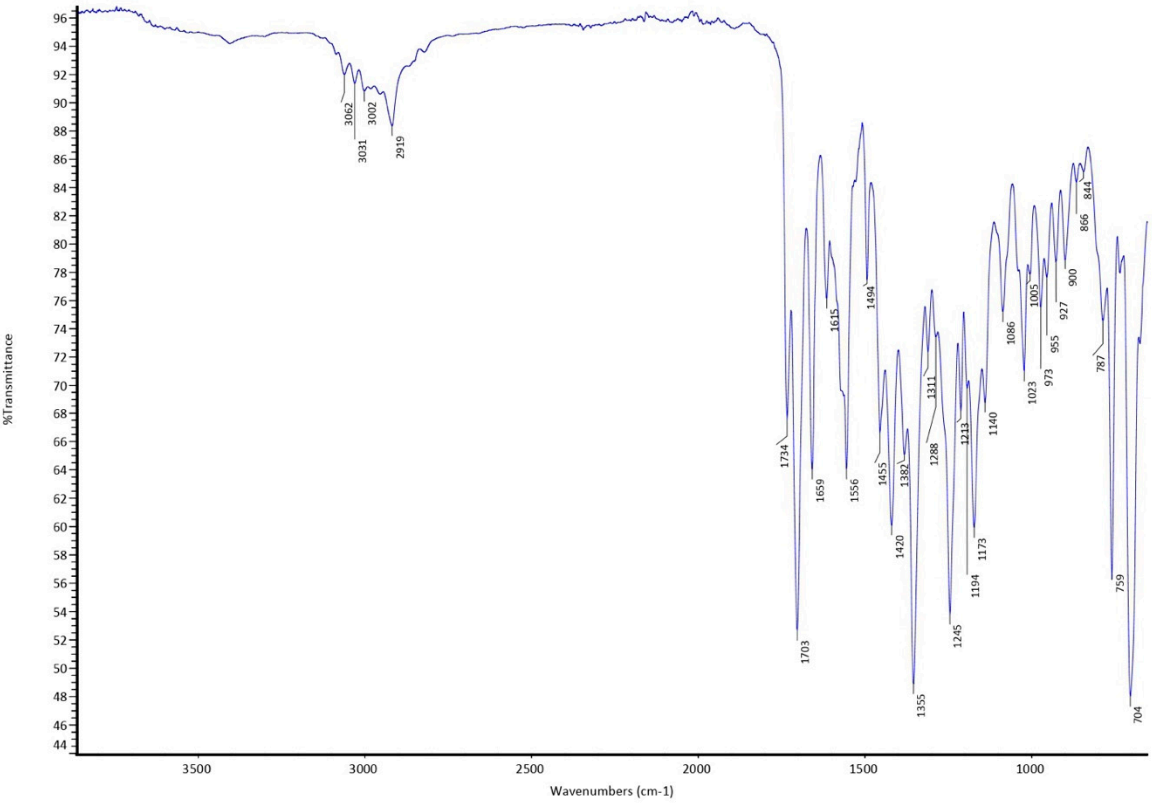

Figure S27. FT-IR spectrum of [Cu(HL^{acPzMe})$_2$(L^{acPzMe})$_2$] (**6**).

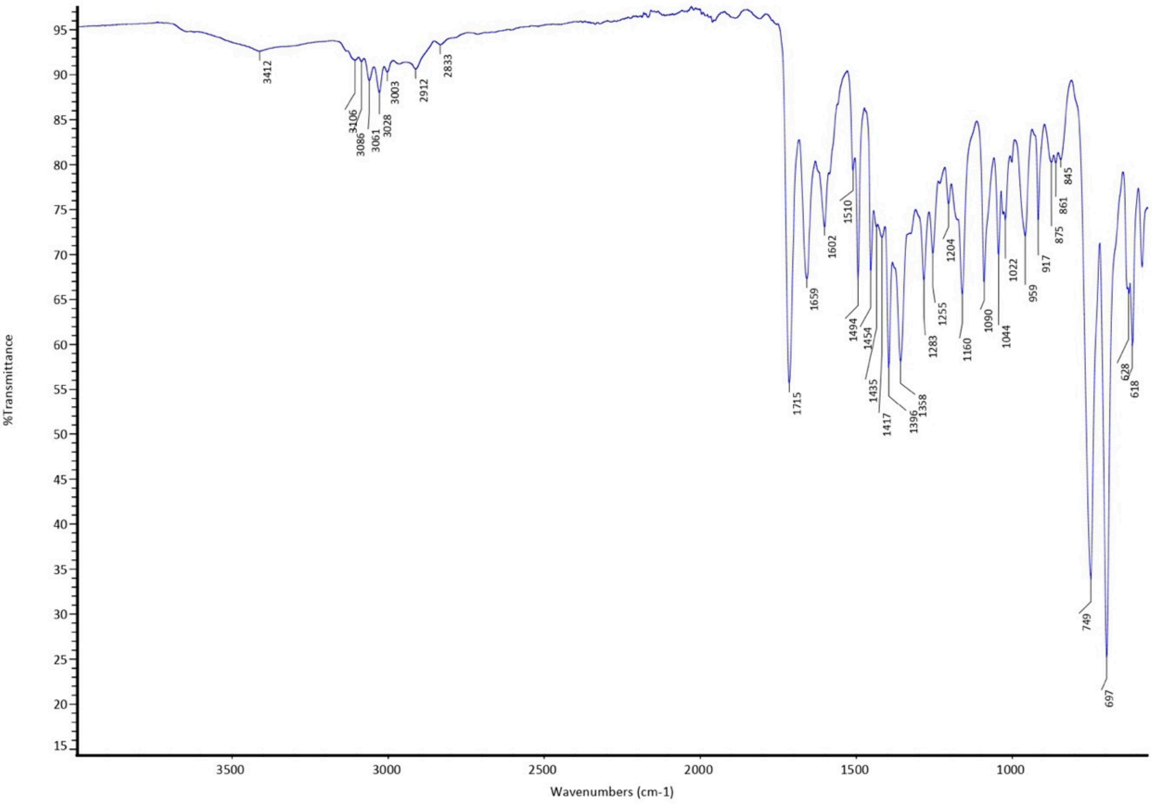

Figure S28. FT-IR spectrum of ^{PhPz}MEK (**7**).

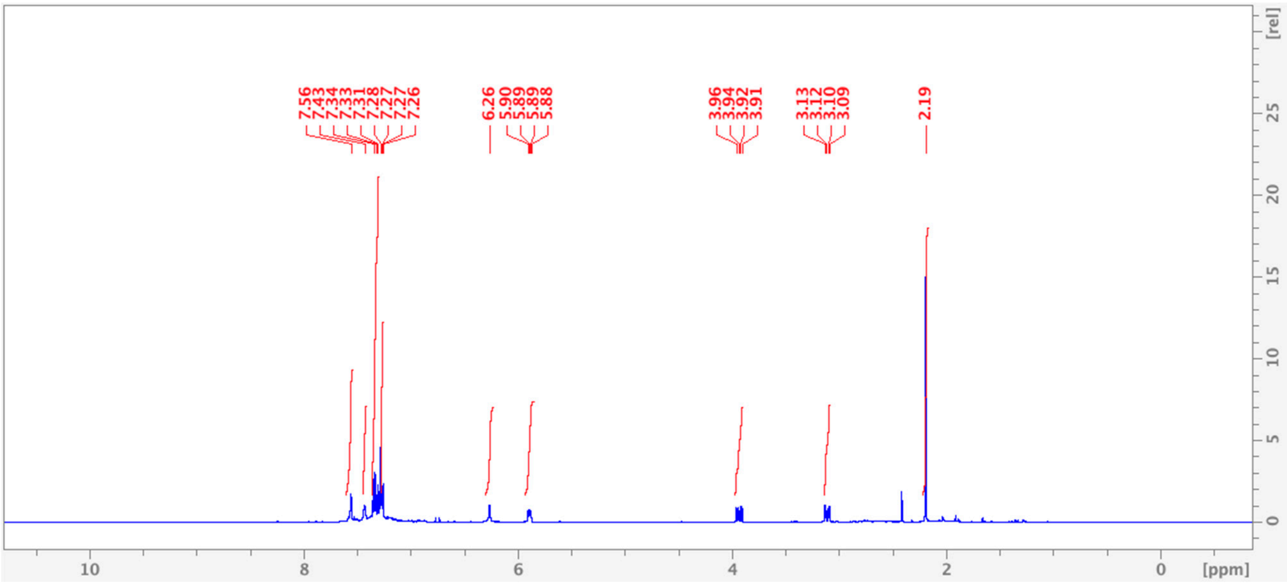

Figure S29. ^{1}H-NMR spectrum of ^{PhPz}MEK (**7**) in $CDCl_3$.

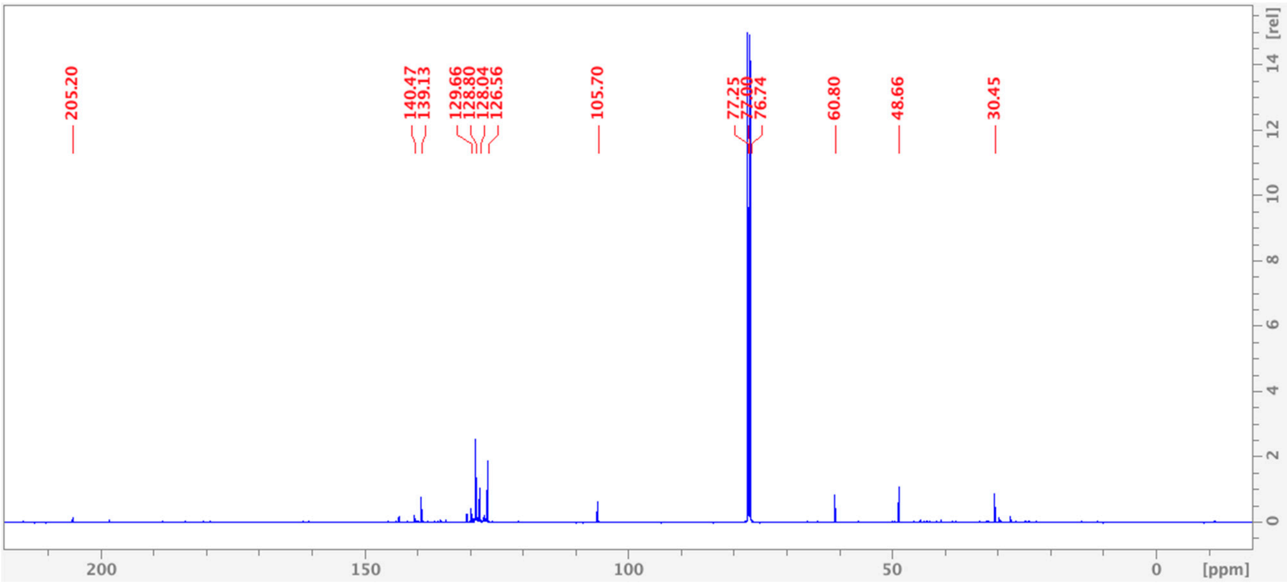

Figure S30. $^{13}C\{^{1}H\}$-NMR spectrum of ^{PhPz}MEK (**7**) in $CDCl_3$.

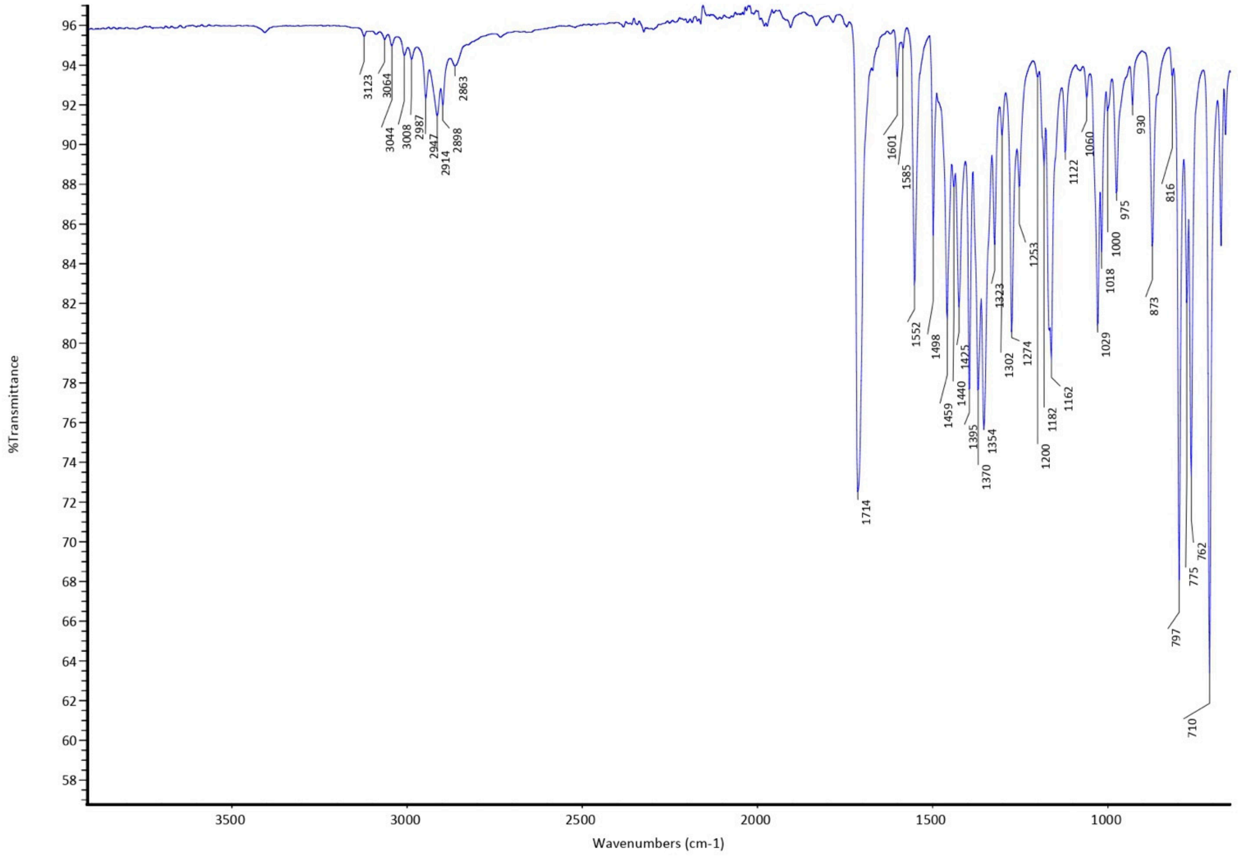

Figure S31. FT-IR spectrum of ^{PhPzMe2}MEK (**8**).

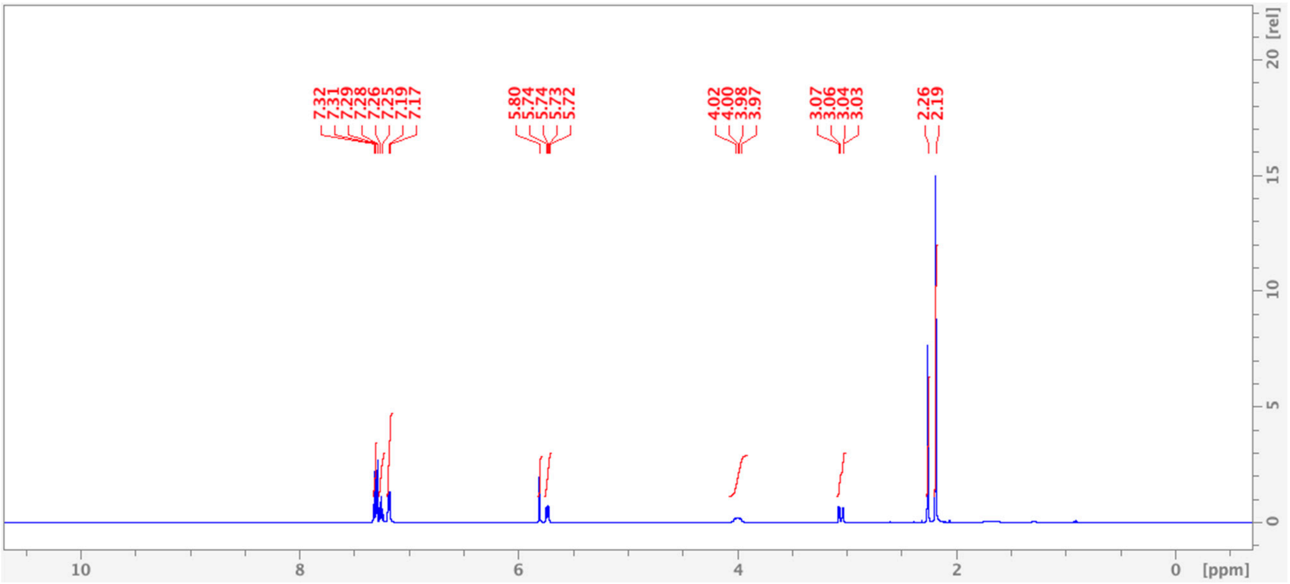

Figure S32. ^{1}H-NMR spectrum of ^{PhPzMe2}MEK (**8**) in $CDCl_3$.

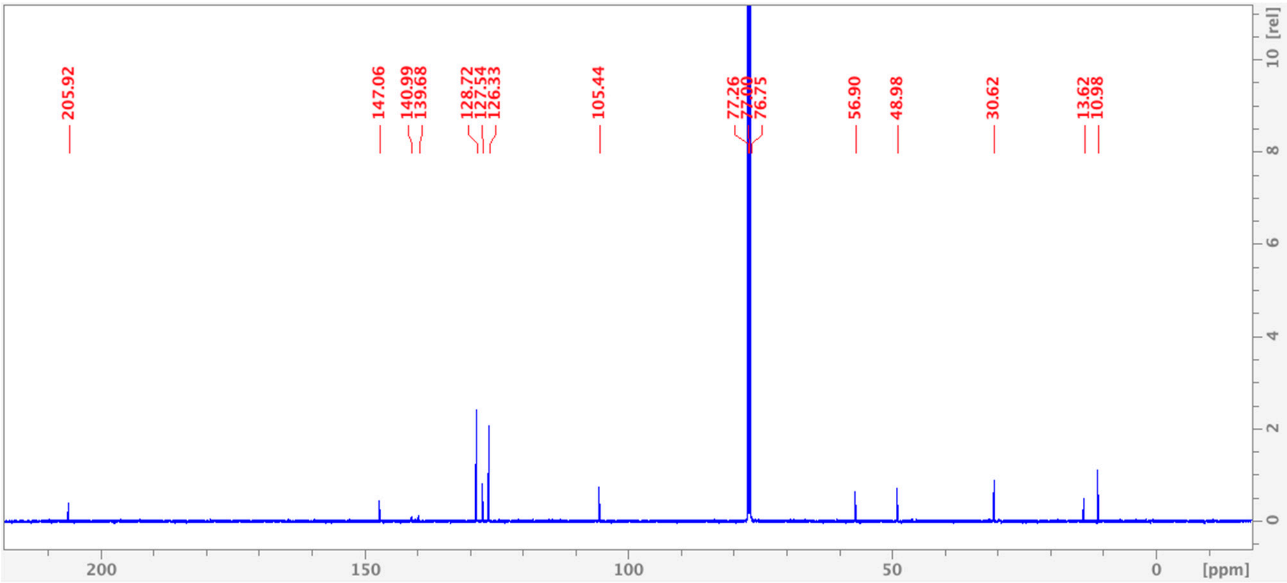

Figure S33. $^{13}C\{^{1}H\}$-NMR spectrum of ^{PhPzMe2}MEK (**8**) in $CDCl_3$.
